# COVID-19: The Emerging Immunopathological Determinants for Recovery or Death

**DOI:** 10.3389/fmicb.2020.588409

**Published:** 2020-12-01

**Authors:** Tanveer Ahmad, Rituparna Chaudhuri, Mohan C. Joshi, Ahmad Almatroudi, Arshad Husain Rahmani, Syed Mansoor Ali

**Affiliations:** ^1^Multidisciplinary Centre for Advanced Research and Studies, Jamia Millia Islamia, New Delhi, India; ^2^Department of Molecular and Cellular Neuroscience, Neurovirology Section, National Brain Research Centre (NBRC), Haryana, India; ^3^Department of Medical Laboratories, College of Applied Medical Science, Qassim University, Buraydah, Saudi Arabia; ^4^Department of Biotechnology, Jamia Millia Islamia, New Delhi, India

**Keywords:** COVID-19, SARS-CoV-2, lymphocytopaenia, T cell response, viral evasion, interferon response

## Abstract

Hyperactivation of the host immune system during infection by SARS-CoV-2 is the leading cause of death in COVID-19 patients. It is also evident that patients who develop mild/moderate symptoms and successfully recover display functional and well-regulated immune response. Whereas a delayed initial interferon response is associated with severe disease outcome and can be the tipping point towards immunopathological deterioration, often preceding death in COVID-19 patients. Further, adaptive immune response during COVID-19 is heterogeneous and poorly understood. At the same time, some studies suggest activated T and B cell response in severe and critically ill patients and the presence of SARS-CoV2-specific antibodies. Thus, understanding this problem and the underlying molecular pathways implicated in host immune function/dysfunction is imperative to devise effective therapeutic interventions. In this comprehensive review, we discuss the emerging immunopathological determinants and the mechanism of virus evasion by the host cell immune system. Using the knowledge gained from previous respiratory viruses and the emerging clinical and molecular findings on SARS-CoV-2, we have tried to provide a holistic understanding of the host innate and adaptive immune response that may determine disease outcome. Considering the critical role of the adaptive immune system during the viral clearance, we have presented the molecular insights of the plausible mechanisms involved in impaired T cell function/dysfunction during various stages of COVID-19.

## Introduction

Following reports of severe pneumonia cases of unknown etiology from Wuhan, China, multiple groups identified the pathogenic agent responsible for the current COVID-19 pandemic as the SARS-CoV-2 virus (Chan et al., 2020; Huang C. et al., 2020; Zhu et al., 2020). The last several months have seen an unprecedented surge in research efforts to understand the underlying molecular mechanisms associated with SARS-CoV-2 infectivity, immunogenicity, and pathogenesis. Since it is now evident that SARS-CoV-2 employs the same set of receptors and host cells, previously utilized by SARS-CoV, various disease models were developed to understand the molecular networks implicated in viral evasion, host immune response, and immuno-pathogenesis. Multiple factors and pathways are already known based on previous knowledge from other coronaviruses, which have shown promising potential as therapeutic targets (Tay et al., 2020). But a more comprehensive understanding to develop highly effective therapies is yet to emerge, which demands better molecular details at various stages of the virus propagation and disease progression in the host cells. In the initial early mild phase of infection (Stage I), the virus remains confined to the upper respiratory tract (nasal cells, some areas of pharynx and larynx) which elicits low levels of the innate immune response (if any). This asymptomatic state lasts for a couple of days (generally one or two days) before the virus propagates to the conducting and terminal airways (Stage II). During this stage of the disease, an optimal but controlled adaptive and innate immune response will help to combat the infection. Successful viral clearance from recovered patients, show the presence of adequate adaptive immune cells along with the immunomodulatory molecules and neutralizing antibodies (Cao, 2020; Tay et al., 2020). However, an impaired adaptive immune response at this stage, with concomitant overactivation of the innate immune system (inflammatory macrophages and neutrophils) can lead to severe disease symptoms in ∼20% of COVID-19 patients (Wu and McGoogan, 2020). Recent clinical and histopathological data from deceased patients suggest adaptive immune dysfunction and heightened proinflammatory response with inflammatory cell infiltration into the lungs (Stage III). Further, the disease severity positively correlated with increased levels of proinflammatory IL-6 and an increase in the neutrophil/lymphocyte ratio (Liu T. et al., 2020; Tan L. et al., 2020b; Tan M. et al., 2020c). Between 3 and 17% of COVID-19 patients developed acute respiratory distress syndrome (ARDS), as a result of hyper inflammation (excessive infiltration of activated innate immune cells and cytokine release syndrome) and lymphocytopenia (reduced levels of CD4+, CD8+, and B cells) (Gibson et al., 2020). These changes are followed by cell death and tissue destruction, which ultimately leads to airway collapse, multiple organ failure and death in 67–85% of ICU patients, based on the available data so far (Wu and McGoogan, 2020; Xu Z. et al., 2020; Zhang H. et al., 2020). Here, we discuss the molecular determinants implicated in the success or failure of recovery through various phases of immune response generated by the host cells. We have built an immunological trajectory of COVID-19 patients who have successfully cleared the virus against those who have developed severe symptoms, with emphasis on virus sensing and evasion mechanisms, and the spatiotemporal role of the innate and adaptive immune system. Further, we provided cellular and molecular details of cytokine storm and ARDS in COVID-19 patients.

## Innate Nucleic Acid Sensing and Viral Evasion Mechanisms

### Nucleic Acid Sensors in Antiviral Signaling

**FIGURE 1:**
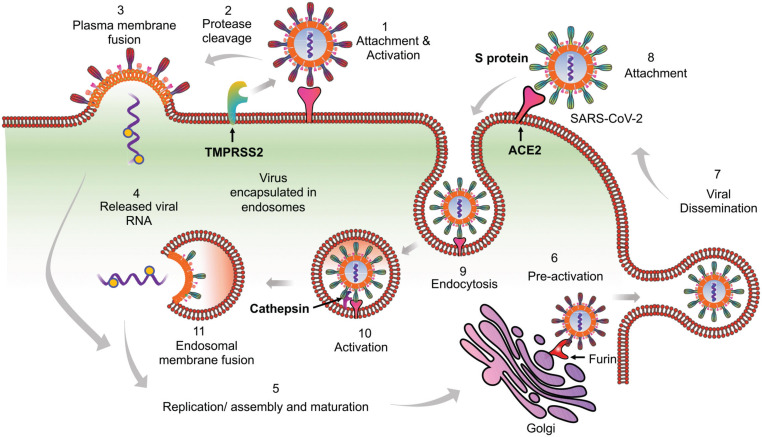
Proposed model of SARS-CoV-2 entry into the host cells. Based on available literature on SARS-CoV and recent findings on SARS-CoV-2, we suggest two different mechanisms that can be employed by SARS-CoV-2 to enter into the ACE2 expressing cells. (1) Initially the virus may use the cell membrane mode of entry. The first step is the binding of the spike protein of the virus with ACE2 receptors expressed on the plasma membrane of host cells. (2) The attachment with ACE2 is followed by the cleavage of S protein by membrane bound proteases like TMPRSS2. TMPRSS2 cleaves the membrane bound virus at both S1/S2 boundary as well as at S2’ site. (3) This activates the fusion machinery, and subsequently, the viral membrane fuses with the host cell plasma membrane. (4) This leads to release of the viral nucleocapsid into the cytoplasm. (5) The replication, assembly, and maturation of virus takes places in the cytoplasm. (6) Before dissemination, SARS-CoV-2 may also undergo pre-activation in the golgi apparatus by furin proteases. (7) The fully mature and pre-activated SARS-CoV-2 eventually disseminates from host cells by exocytosis. During subsequent infection cycles, the virus may utilize either cell membrane or (8–11) the more probable endocytic entry route. In the endocytic mode of entry, (8) after attachment with ACE2, (9) the virus gets endocytosed and (10) then processed at the S2’ region by endosomal proteases like cathepsins, to activate membrane fusion. (11) Finally, the viral components are released into the cytoplasm by fusion of the viral membranes with endosomal membrane, leading to repeat of the cycle.

SARS-CoV-2, like its predecessor SARS-CoV, employs spike (S) protein to enter into the eukaryotic cells by binding to the surface-expressed ACE2 receptors. Upon binding, S protein priming takes place by the membrane expressed protease TMPRSS2 or endosomal proteases such as cathepsin, elastase, and furin (which is specific to SARS-CoV-2) to induce fusion between the viral and host cell membrane (Hoffmann et al., 2020; Shang et al., 2020; Walls et al., 2020; Wang Q. et al., 2020). Following these well-coordinated events, viral genetic material will release in a biphasic manner, i.e. either by direct fusion with the plasma membrane or by following the endocytic route as shown previously for SARS-CoV (Belouzard et al., 2012; Shang et al., 2020; Figure 1). An increasing list of cell types appear directly infected by the SARS-CoV-2, which include the alveolar epithelial type II cell (ATII) as the principal targets, and other cell types lining various tissues such as bronchial epithelial cells in lungs, goblet cells in the nasal cavity, macrophages, esophageal cells, pancreatic β-cells, and gastrointestinal epithelial cells (Li M.Y. et al., 2020; Sungnak et al., 2020). All these cell types express the S protein target receptor ACE2, albeit with lower expression. However, ATII cells remain the predominant targets for SARS-CoV-2 as for SARS-CoV, which are involved in the sensing of the various viral proteins.

Preceding studies on human infecting coronaviruses (CoVs) have demonstrated a critical role of nucleic acid-sensing (NAS) pathways in recognizing various components of these viruses to initiate an early antiviral response. Whereas, potent inhibitory mechanisms are developed by CoVs to prevent or delay early antiviral responses (Rose et al., 2010; Adedeji et al., 2013). These inhibitory signals affect a range of host defense pathways to allow the propagation of CoVs. Some inhibitory signals may even activate cell death pathway to induce a robust proinflammatory state. Studies from in vitro cell culture, animal models, and patients who have successfully recovered from SARS-CoV infection have provided detailed molecular insights about signaling molecules implicated in virus-host interaction that may also serve as a model to understand a similar process in SARS-CoV-2 (Totura and Baric, 2012).

After release into the cytoplasm, the ssRNA viral genomes of SARS-CoV and SARS-CoV-2 proceed to replication via a double-stranded RNA (dsRNA) intermediate state (Adedeji et al., 2012; Cascella et al., 2020). Both ssRNA and dsRNA act as pathogen-associated molecular patterns (PAMPs) which are recognized by pathogen recognition receptors (PRRs; Leiva-Juárez et al., 2018). ATII cells are known to express key endogenous PRRs like Toll-like receptors (TLRs), cyclic GMP–AMP synthase (cGAS); and retinoic acid-inducible gene I (RIG-I)-like receptors (RLRs). Among these, cytosolic RLRs and endosomal TLRs (TLR3, TLR7, TLR8, TLR9) have a prominent role in initiating the antiviral response by sensing RNA from SARS-CoVs (Lester and Li, 2014; Chan and Gack, 2016).

**FIGURE 2:**
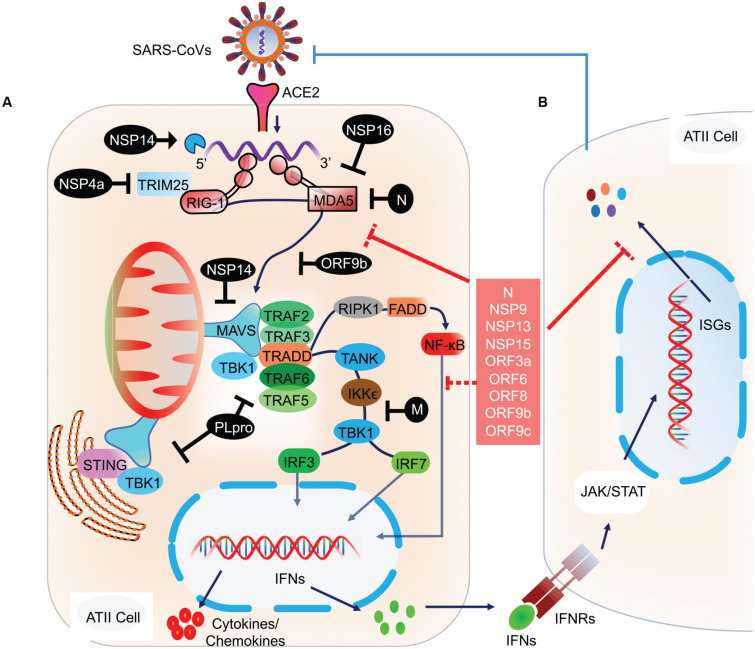
Molecular and signaling pathway implicated in host cell antiviral response. **(A)** After the viral contents are released into the cytoplasm, the viral RNA is recognized by host cell NASs like RIG-I and MDA5. Counter-defense may be provided by the viral proteins, NSP14 and NSP16 to shield the viral RNA from sensing by the NASs. However, if successfully recognized, RIG-I and MDA5 get activated and subsequently activate the centrally placed MAVS located on mitochondria. MAVS acts as a molecular adaptor that further recruits TRAF2/3/5/6. Association of the type of TRAF with MAVS is suggested to determine the type of downstream signaling, i.e., IRF3/7 and/or NF-κB. At the MAVS junction, the association of TRAF5/6 with TRADD, FADD, and RIPK1 activates NF-κB. Whereas, binding of MAVS with STING activates TBK1 and IKKε by interacting with TRAF2/3, which eventually results in the activation of IRF3 and IRF7 (Chen et al., 2014). Activated IRF3, IRF7, and NF-κB translocate to the nucleus and induce the expression of IFN genes. **(B)** The transcribed IFNs act on the respective IFN receptors (IFNRs) present on the host cells as well as on other innate immune cells, thus signaling in a both autocrine and paracrine manner. Signaling via IFNRs activates the JAK/STAT signaling pathway and subsequently induces the expression of ISGs. These molecular events were recently reviewed (Rehwinkel and Gack, 2020). ISGs transcribed will eventually inhibit viral propagation. However, SARS-CoV and likely SARS-CoV-2 have developed counter-defense mechanisms to interfere at various steps in the NAS signaling pathway. NSP4a inhibits TRIM25, which is required for RIG-I activation. N protein inhibits MDA5, NSP14 inhibits MAVS, ORF9b inhibits RIG-I/MDA5 activation complex, M protein interferes with TANK, IKKε, and TBK1 signaling, and PLpro inhibits various RIG-I, MDA5, and MAVs downstream signaling steps. SARS-CoV-2 proteins acting at various steps in blocking NAS and IFN signaling are shown in the red box. NAS, Nucleic acid sensors; RIG-I, Retinoic acid-inducible gene I; MDA5, melanoma differentiation-associated protein 5; TRAF, TNF receptor-associated factor; STING, ER-associated stimulator of interferon genes; FADD, FAS-associated death domain protein; IRF, Interferon regulatory factor (IRF3/7); TRADD, TNFR1-associated death domain protein; IKKε, IκB kinase-ε; RIPK1, Receptor-interacting protein 1; TANK, TRAF family member-associated NF-kappa-B activator; TBK1, TANK-binding kinase 1; ISG, Interferon stimulatory gene; TRIM25, Tripartite motif-containing protein 25.

RLRs are a complex of sensor proteins that include RIG-I, melanoma differentiation-associated gene 5 (MDA5), and the more recently discovered probable ATP-dependent RNA helicase DHX58 (also known as LGP2) (Jiang et al., 2011; Leiva-Juárez et al., 2018). RIG-I binds to 5’-PPP RNA and short dsRNA, while MDA5 binds to longer RNA fragments (Huang et al., 2014). Binding of pathogenic RNAs induces conformational changes in RIG-I and MDA5, and after that post-translational modifications activate these proteins. Importantly, RIG-I is activated by E3 ligase tripartite motif protein 25 (TRIM25) via polyubiquitination at K172 residue (Sanchez et al., 2016); MDA5 is proteolytically inactivated by the polyubiquitination mediated by poly (rC) binding protein 2 (PCBP2) with assistance from AIP4/ITCH (Atrophin 1 Interacting Protein 4; also called ITCH) (You et al., 2009). LGP2 acts as a facilitator to enhance viral sensing by RIG-1 and MDA5 (Satoh et al., 2010). Activated RIG-I and MDA5 then mount the downstream signaling cascade via centrally placed mitochondrial antiviral signaling protein (MAVS) and eventually lead to the coordinated activation of IRF3/IRF7 transcription factors (Figure 2). Activated IRF3/7 translocates to the nucleus and induces expression of IFNs via IFN-stimulated response element (ISRE) reviewed by West et al. (2011) and Rehwinkel and Gack (2020). Thus, centrally placed MAVS activation induces expression of IFN genes via IRF3 and IRF7 pathways and recruitment of other innate immune cells, majorly by proinflammatory molecules secreted via NF κB signaling (Figure 2). Similarly, activation of endogenous TLR pathway induces expression of IFN type I, type III, and more specifically, proinflammatory molecules via the NF κB pathway (Gong et al., 2020). Blocking of either IRF3/7 or NF κB pathway has a detrimental effect on host cells that invariably allows propagation of the virus (Lazear et al., 2013; Schmitz et al., 2014; Totura et al., 2015; Chiang and Liu, 2019). In animal studies, mice that are deficient in TLR signaling exhibit robust infection and severe pathological condition during SARS-CoV infection. TLR3 and TLR4 knockout mice exhibited increased viral titers associated with lung damage and a higher mortality rate (Totura et al., 2015). Mice with a knockout of myeloid differentiation primary response 88 (MYD88), which acts downstream of TLR signaling had increased damage to the lung parenchyma with a 90% mortality rate (Sheahan et al., 2008). Conversely, activation of endogenous TLR signaling by TLR7, TLR8, and TLR9 or cell surface-expressed TLR4 signaling was associated with a significant decrease in viral propagation, attenuated lung damage, and increased the survival rate in SARS-CoV infected animals (Zhao et al., 2012). These findings thus point to an integral role of these molecular sensors in mounting early protective antiviral response and aiding viral clearance.

The role of these molecular sensors is not yet comprehensively studied in SARS-CoV-2, but a few recent reports suggest that these sensors are similarly involved in the early antiviral response during infection. The immunoinformatic approach revealed the presence of a wide range of ssRNA SARS-CoV-2 genome fragments as potential molecular PAMPs which were presumed to mediate signaling via endogenous TLR7/8 pathway. Further, it is appearing that the number of PAMPs (genomic fragments) was higher in the SARS-CoV-2 genome as compared to SARS-CoV, suggesting that SARS-CoV-2 may drive relatively more robust immune response (Moreno-Eutimio et al., 2020). Single-cell RNA-sequencing (scRNA-seq) study in PBMCs derived from ICU patients revealed extensive upregulation of NAS pathway genes including RIG-I, MDA5, and LGP2, suggesting an invasion of SARS-CoV-2 in these cells (Wei et al., 2020). However, no direct assays were performed in these cells to find the presence or absence of viral RNA. These findings may imply that that the SARS-CoV-2, does not directly infect PBMCs and thus this upregulation of NAS genes may be through passive uptake of the virus, most probably by antibody-dependent enhancement (ADE), as will be discussed later. Similarly, endogenous TLR7 and TLR8 upregulate along with an increase in expression of MAVS, IRF3, and IRF7. The functional importance of this upregulated expression of NAS pathway genes remains unclear and hence more research in this direction will clarify the specific role of these molecular sensors in the antiviral response against SARS-CoV-2.

### Evasions Mechanism Employed by SARS-CoVs

All human infecting SARS-CoVs are known to have evolved multiple mechanisms to evade recognition by host cells. Emerging evidence suggests that similar mechanisms are employed by SARS-CoV-2 to inhibit or delay the host cell immune response. Some of these mechanisms will be discussed below.

#### Interference With the Nucleic Acid Sensing and Downstream Signaling

Previous studies on SARS-CoV revealed smart strategies to inhibit multiple steps in the NAS pathway and downstream signaling (Rose et al., 2010; Adedeji et al., 2013; Chan and Gack, 2016). As mentioned earlier, TRIM25 mediated ubiquitination activates RIG-I. Whereas, the N protein of SARS-CoV, which binds to TRIM25 and thereby prevents its association with RIG-I and hence activation. The ubiquitin usurped RIG-I is unable to mount the antiviral response, thereby disabling IFN-β production (Hu et al., 2017). N protein also antagonizes IFN signaling by directly interacting with IRF3, thereby inhibiting its phosphorylation and subsequent nuclear translocation (Kopecky-Bromberg et al., 2006; Kopecky-Bromberg et al., 2007). Similarly, M protein inhibits IRF3/IRF7 signaling by interfering with RIG-I, TBK1, IKKε, and TRAF3 activation complex formation (Siu et al., 2009). Acting at multiple pathways on host cells, Nsp1 inhibits IFN-β promoter activity and STAT1 phosphorylation which led to a decrease in the expression of various antiviral interferon-stimulated genes (ISGs; Wathelet et al., 2007). Chen et al. (2014) showed that papain-like protease (PLpro) directly associates with TRAF3, TBK1, IKKε, STING, and IRF3 and hence inhibits downstream IRF3/IRF7 signaling. In another study, Devaraj et al. (2007) showed that PLpro inhibits IRF3 phosphorylation and its subsequent nuclear translocation. ORF3b, ORF6, ORF8a, and ORF8b also play prominent roles in inhibiting IRF3 phosphorylation and its subsequent nuclear translocation (Kopecky-Bromberg et al., 2006; Freundt et al., 2010; Wong et al., 2018). ORF9b was shown to be associated with mitochondria and induced degradation of dynamin-related protein 1 (Drp1), thus altering mitochondrial function and sequestering MAVS into small puncta. Further, ORF9b was associated with recruitment of ubiquitin ligases PCBP2 and AIP4 E3 which led to ubiquitination of MAVS and eventually its degradation, as a result inhibiting IFN-β production (Shi et al., 2014). Thus, by associating with multiple proteins involved in NAS signaling, SARS-CoV antagonizes IFN signaling and synthesis of protective molecules like ISGs.

Recent studies have also demonstrated the interaction of SARS-CoV-2 proteins with multiple host cell NAS signaling molecules and downstream IFN signaling. An extensive proteomic study by Gordon et al. (2020), showed multiple SARS-CoV-2 protein and host cell protein interactions. A proteome map of 26 SARS-CoV-2 proteins predicted 332 viral proteins interacting with host cells. Among these, Nsp9, Nsp13, Nsp15, ORF3a, ORF9b, and ORF9c interacted with proteins in downstream NAS signaling, IFN response, and NF-κB pathway. Similarly, Nsp5 interacted with HDAC2, which may be thus involved in limiting the IFN signaling and inflammatory response, but the specific functional role of these proteins was not determined (Gordon et al., 2020). In two recent studies, the functional relevance of some of these proteins was tested in vitro. In the first study, Li J.Y. et al. (2020) tested the effects of ORF6, ORF8 and N protein on the antiviral response in HEK293 cells and found these proteins inhibit IFN-β and NF-κB signaling. Similarly, Yuen et al. (2020) showed that IFN antagonizing effect of ORF6 was due to its association with the interferon-inducible nuclear export complex (NUP98–RAE1). The study further showed that Nsp13, Nsp14, and Nsp15 could also antagonize IFN response, but the mechanism was not explored (Yuen et al., 2020).

In addition to interfering with IFN production pathway, SARS-CoV has evolved multiple other mechanisms to modify host cell response. Viral RNA is unprotected and open to cellular degradation; however, some RNA viruses have evolved a capping process to evade recognition by the host. In SARS-CoV, Nsp16 provides ribose 2′-*O*-methylation at the 5′ end of the RNA to protect its degradation and prevent sensing by MDA5 (Züst et al., 2011). Similarly, Nsp14 had N7 methyltransferase activity and methylated the 5′ end of the RNA (Chen et al., 2009). Other SARS-CoV proteins involved include – Nsp4a, which prevents stress granule formation by inhibiting PKR mediated antiviral signaling (Rabouw et al., 2016). N protein of SARS-CoV-2 is also known to interact with the proteins implicated in stress granule regulation (Gordon et al., 2020). Electron tomography studies in SARS-CoV infected cells revealed a unique replication network derived from ER to organize viral replication while simultaneously hiding the viral RNA from recognition by host NASs (Knoops et al., 2008). Other RNA viruses have also developed similar strategies to evade sensing by forming double-membrane vesicles (DMVs) and replication organelles to prevent access to the NASs (Blanchard and Roingeard, 2015).

#### Inhibition of Host Cell Biosynthetic Pathways and Modulation of Cell Death

Both SARS-CoV and SARS-CoV-2 have evolved multiple inhibitory mechanisms to evade host cell recognition. Inhibition of host transcriptional and translational machinery prevents the biosynthesis of protective IFNs and delays early activation of host cell apoptosis. Nsp1 of SARS-CoV inhibit the loading of ribosomal 40s subunit and prevent host cell protein translation. Further, Nsp1 specifically degrade host cell RNA while sparing the viral RNA (Huang et al., 2011; Tanaka et al., 2012; Lokugamage et al., 2015). N protein of SARS-CoV-2 also interacts with the host biosynthetic protein La-related protein 1 (LARP1). This interaction may serve as the necessary signal to shut down the host cell protein synthesis for the propagation of SARS-CoV-2 (Gordon et al., 2020).

Papain-like protease of SARS-CoV directly interacts with p53 and induce its degradation, which may thus interfere with translation and delay early apoptosis of the infected cells (Yuan et al., 2015; Ma-Lauer et al., 2016). SARS-CoV S protein also interacts with the translation initiation factor eIF3f and inhibit host cell translation by preventing its nuclear import (Xiao et al., 2008). Studies from other respiratory viruses have shown that cells which activate early apoptosis prevent further spread of the viruses, whereas viruses that successfully inhibit this pathway exhibit strong infectivity (Orzalli and Kagan, 2017). Cytomegaloviruses (CMVs) distinctly rely on this mechanism to successfully replicate within the host cell by inhibiting apoptosis-modulatory proteins such as Bax and Bcl-2 (Çam et al., 2010). However, whether SARS-CoV or SARS-CoV-2 are also directly involved in inhibiting early apoptosis remains to be tested, but it is evident that these viruses induce host cell death after successful propagation and dissemination.

SARS-CoV Nsp7a was shown to interact with prosurvival protein Bcl-X and induce apoptosis in cells in vitro (Tan et al., 2007). Similarly, ORF3a leads to fragmentation of the Golgi apparatus, and induction of apoptosis (Waye et al., 2005; Freundt et al., 2010). Besides this, ORF3a also implicates necroptotic cell death by interacting with and activating the main necroptosis protein RIPK3 (Yue et al., 2018). Owing to its role in cell death pathways, the ORF3a of SARS-CoV-2 was also explored in this context. This protein similarly induced apoptosis in HEK293 cells by activating the caspase 8-dependent pathway (Ren et al., 2020).

Interestingly, the results, that ORF3a of SARS-CoV-2 induces relatively lower apoptosis in several cell lines as compared to SARS-CoV, suggesting that this mechanism could provide an early advantage for the propagation of SARS-CoV-2. Further, the proteome map of SARS-CoV-2 predicted interaction of Nsp12 with RIPK1, suggesting that this viral protein may also implicate in regulating host cell apoptotic and necroptotic cell death (Gordon et al., 2020). However, a study on 25 cell lines in culture showed SARS-CoV-2 exhibiting cytopathic effect on only two cells, indicating that the differences could exist between these two related viruses in their property to interfere with host cell death pathways (Chu et al., 2020). Thus, more comprehensive studies are needed to provide better molecular insights by which SARS-CoV-2 modulates host cell death pathways, which may also open new opportunities for treatment.

Based on these early observations, it is becoming evident that SARS-CoV-2 interferes with host NAS, IFN, biosynthetic, and cell death pathways to prevent early immune response and thus contribute to the underlying immunopathogenesis, as will be discussed subsequently. To make these details simple, here we compiled the role of various SARS-CoV and SARS-CoV-2 proteins and their host cell interacting proteins and presented in the Table form (Supplementary Table 1).

## Innate Immune Response in COVID-19

### Functional Innate Immune Response

A balance between successful evasion of the virus from host cell sensing pathways and the counter mechanisms developed by the host cells to overcome these inhibitory effects determines whether an early immune response could be generated or not (Liang et al., 2020). Though most of the studies point towards the successful evasion mechanisms employed by CoVs, emerging evidence suggests that an adequate early antiviral response could be mounted (Park and Iwasaki, 2020). That early response may hold the key for limiting the viral propagation in the majority of the COVID-19 patients (approx 80%) who are asymptomatic or develop mild symptoms and successfully clear the virus. Considered the recent work on COVID-19, here we provide a detailed molecular and clinical understanding of the innate immune response. We specifically discuss how these immune responses dictate the recovery from disease or development of the immunopathological state.

#### Interferon Response

By initiating an early antiviral response, signaling via IFNs and ISGs is critical for the viral clearance and an impediment for the development of the pathological state. Several in vitro and animal studies have established the central role of these signaling pathways in SARS-CoV infection. STAT1 knockout mice infected with SARS-CoV exhibited severe disease symptoms, conferred by increased viral replication and propagation and was further associated with reduced survival rate (Hogan et al., 2004; Frieman et al., 2010). Similarly, SARS-CoV propagation increases in IFNR1-/- and ILFNLR1/- double knockout mice, suggesting an essential role of these signaling pathways in mitigating antiviral response (Mahlakõiv et al., 2012).

Recent in vitro studies point to a more robust IFN response generated by SARS-CoV-2 compared to its predecessor. Epithelial cells infected with SARS-CoV-2 displayed better IFN response than cells infected with SARS-CoV. This IFN response was STAT1 phosphorylation-dependent with subsequent expression of antiviral ISGs (Lokugamage et al., 2020). In line with these in vitro findings, transcriptome data from bronchial alveolar lavage fluid (BALF) taken from 8 COVID-19 patients revealed extensive upregulation of about 83 ISGs, suggesting robust IFN response generated against SARS-CoV-2 (Zhou Z. et al., 2020). Further, a study by Ziegler et al. (2020) suggested that ACE2 may also act as a type of ISG in some respiratory epithelial cells; this may point towards using ACE2 modulators as viable therapeutic options for SARS-CoV-2.

Based on the recent clinical data on COVID-19 patients, we can infer that mild/moderate patients should possess optimal early IFN response. Whereas, weak or delayed IFN response may be the tipping point in eliciting hyperinflammatory state, allowing extensive viral propagation. Previous studies in animal models have shown that early IFN response was the determining factor in inhibiting viral propagation and attenuating disease condition (Channappanavar et al., 2016). In line with this, a recent study has shown that COVID-19 patients with mild/moderate conditions possess functional type I and type III IFN response. Specifically, patients with mild/moderate symptoms have adequate levels of IFNA transcript and protein in the plasma. The presence of detectable IFN levels in these subsets of patients was also associated with the expression of downstream signaling receptors and molecules like IFNAR1, JAK1, and TYK2, suggesting functional IFN response. However, no IFNB mRNA or protein was detected, while optimal levels of IFN-λ were detected both at the mRNA and protein levels. Expectedly, the levels of type I and type III IFNs positively correlated with the viral load and severity of the disease (Hadjadj et al., 2020).

In agreement with the critical role of early IFN response in attenuating infectious state, another study finds that cells pre-treated with IFN-β or IFN-λ exhibit resistance to SARS-CoV-2 infection by significantly decreasing the virus copy number. Similarly, 3D culture organoids pre-treated with either IFN-β or IFN-λ led to reduced viral infection. Cells depleted for either IFNAR1 or IFNLR1 had an overall increase in the number of SARS-CoV-2 infected cells, suggesting the integral role of IFN signaling in attenuating viral propagation (Stanifer et al., 2020). Further, IFN response was adequate in younger patients compared to older ones, which may partly explain the higher risk of infection in older people (Wei et al., 2020). Additionally, people with comorbid conditions like diabetes – a condition associated with impaired IFN response, are more susceptible to SARS-CoV-2 infection, which further points toward the critical role of IFN signaling in the early clearance of the virus (Erener, 2020). However, a comprehensive and longitudinal analysis of the IFN response in mild/moderate patients is warranted to understand the functional consequence of this immune response throughout the disease and recovery. Overall, considering the relatively better IFN response and ISG expression induced by SARS-CoV-2, one can argue that this functional immune response is a probable reason for the relatively lower mortality rate seen in COVID-19, compared to previous SARS-CoV and MERS infections (Meo et al., 2020). However, these early findings warrant further proof.

#### Early Immune Response by Alveolar Epithelial Cells (ATII)

Activated alveolar macrophages (AM) and recruited inflammatory monocytes/macrophages are majorly responsible for the secretion of cytokines and chemokines in early phases of infection, with a substantial contribution from infected ATII cells as well. This early response is necessary to recruit and activate the adaptive immune system and hence drive the clearance of the virus without inflicting immunopathological state. While the levels of cytokines and chemokines are well-regulated during this phase of infection, a check on the activation profile and recruitment of these innate immune to the sites of infection is critical. Thus, a regulated and controlled release of cytokines and chemokines in the early phase of infection is not necessarily proinflammatory but drives the successful viral clearance and the probable reason behind the limited propagation of infection as seen in the majority of the COVID-19 cases exhibiting mild symptoms (Song et al., 2020; Tay et al., 2020).

Among the cytokines secreted by virus-infected airway epithelial cells, IL-6 plays a prominent role in the early recruitment and differentiation of monocytes, neutrophils, and lymphocytes which express the corresponding IL-6 receptor (IL-6R). Though IL-6 is chiefly secreted by macrophages (activated AMs and inflammatory macrophages) in the lungs, secretion of IL-6 by ATII is also significant. In vitro studies on SARS-CoV have shown the release of IL-6 by ATII in response to RIG-I and TLR signaling via activation of NF κB pathway (Ndlovu et al., 2009; Tanaka et al., 2012). Additionally, proinflammatory cytokines TNF-α and IL-1β secreted by macrophages act on ATII cells to cause the release of IL-6 (Crestani et al., 1994; Schwingshackl et al., 2013).

Transcriptional profiling in normal human bronchial epithelial (NHBE) infected with SARS-CoV-2 shows upregulation of IL-6, suggesting that these lung epithelial cells may contribute to early IL-6 response seen in non-severe COVID-19 patients (Blanco-Melo et al., 2020). However, more conclusive studies like tissue immunohistochemistry or single-cell immuno-profiling of the lung epithelial cells will clarify their contribution in IL-6 secretion *in vivo*.

#### Early Immune Response by Alveolar Macrophages

Lung resident macrophages like AM are generally present in the terminal airways where they serve a regulatory function to maintain normal cellular homeostasis. Previous studies have defined a critical role of these cells in successful viral clearance (Hartwig et al., 2014). Depletion of these cells in animals infected with mouse hepatitis virus type 1 (MHV-1) resulted in a marked reduction of antiviral response. AMs have also been shown indispensable during SARS-CoV infection. The depletion of these cells was associated with worsened disease outcomes in a mouse model of SARS-CoV (Page et al., 2012).

Further, BALF fluid analysis of SARS-CoV infected patients revealed an increase in AM population, which persisted over two months and significantly correlated with viral clearance (Wang et al., 2005). In addition to their activation by the secondary response during viral infection, few in vitro studies have shown that these cells can also be directly targeted by SARS-CoV (Mossel et al., 2008; Joel Funk et al., 2012), though contradictory reports are available (Yip et al., 2014). Overall, the data supporting the antiviral response by AMs cells is largely based on other respiratory infections like influenza virus and MERS, with a few reports on SARS-CoV (Mossel et al., 2008; Joel Funk et al., 2012).

Studying these responses in COVID-19 patients may be challenging due to technical limitations (like difficulty in obtaining the optimal number of these cells from the lungs and their rapid functional and phenotypic changes during cell culture). However, we can draw inferences from other cell types and correlate specific markers from cells directly obtained from the lung tissue. One such recent elegant study using scRNA-seq and cluster analysis revealed the activation status of AMs in BALF fluid derived from COVID-19 patients. The analysis is based on the signature genes expressed by these cells, which are markedly different from recruited inflammatory macrophages (Liao et al., 2020). Surprisingly, the number of these cells declined in patients with severe disease symptoms, and the presence of proinflammatory macrophages can take their place (Liao et al., 2020).

A recent study (pre-print, not yet peer-reviewed) has shown infection and propagation of SARS-CoV-2 in macrophages present in lymph nodes and spleen (Chen Y. et al., 2020). However, direct infection and replication of the virus was not explored in detail, specifically under in vitro settings. Previous studies on SARS-CoV suggest low replication in these cells, probably due to phagocytosis (Yilla et al., 2005). Thus, these results suggest that AMs’ response to SARS-CoV-2 may be complicated but necessary for the activation and recruitment of other innate cells like monocytes, dendritic cells, neutrophils, natural killer (NK) cells, and essential in the regulation of the adaptive immune system (Soroosh et al., 2013; Hartwig et al., 2014; Meischel et al., 2020).

#### Dysfunctional Innate Immune Response

On average, about 15% of the COVID-19 patients exhibit severe disease symptoms whereas 5% become critical, but the figures are subject to change owing to the ongoing increase in the number of cases (Berlin et al., 2020). By looking at the immunological trajectories of these patients, it has become evident that impaired early IFN response followed by hyperactivated innate and a dysfunctional adaptive immune response is the vital pathological factors contributing to disease severity in COVID-19 patients (Blanco-Melo et al., 2020; Mathew et al., 2020). However, there are also reports, suggesting a more complex interplay in these immune responses, which needs a thorough understanding of developing effective immunotherapy-based interventions and for successful vaccine development.

#### Impaired Interferon Response

Based on previous molecular and clinical studies on SARS-CoV and the recent data on SARS-CoV-2, it is becoming evident that the delay in primary IFN response may be due to multiple factors such as (1) poor overall immune function of a patient with a compromised adaptive response as in older people, (2) patients with comorbidity, (3) genetic factors or epigenetic changes associated with crucial genes and transcriptional factors involved in IFN signaling, and (4) age and sex of the patient, probably making the older individuals and males more susceptible to COVID-19 (Bastard et al., 2020; Li M.Y. et al., 2020; Nguyen et al., 2020; Verdecchia et al., 2020; Zhou F. et al., 2020). Thus, overall these factors may compromise the host cell immune system and delay the early antiviral response. Especially in the case of RNA viruses, evasion of host immune response is managed by interfering with PRRs, PLRs, TLRs, and IFN signaling (Kikkert, 2020). Additionally, inhibition is also conferred by hijacking host cell biosynthetic machinery and eventually inducing host cell apoptosis as discussed above.

Previous studies have unequivocally demonstrated poor IFN response to SARS-CoV during severe infection, which is also apparently the case with SARS-CoV-2, reviewed recently by Park and Iwasaki (2020). *In vitro* culture of the primary lung, epithelial cells infected with the SARS-CoV-2 generated inadequate IFN response (Blanco-Melo et al., 2020). By looking at the clinical samples, a large body of data suggests impaired IFN signaling in severe and critically ill COVID-19 patients. Blood analysis from across the studies reveals low or undetectable levels of IFN-β and IFN-λ levels in patients exhibiting severe disease symptoms or patients admitted to the ICU with in a critical condition (Hadjadj et al., 2020). Of note, an elegant study was conducted to explore the functional role of IFN signaling during various stages of COVID-19 disease severity. The study found robust impairment of IFN signaling in critically ill and severe patients in comparison to mild/moderate and healthy individuals. IFN-β mRNA and protein were undetectable in all patients, whereas IFN-α2 protein was highly reduced in the plasma of severe and critically ill patients, corroborated with reduced IFN activity. In line with the impaired IFN signaling, robust downregulation of some of the ISGs (MX1, IFITM1, IFIT2) observed in severe and critically ill patients suggest an overall reduced IFN response (Hadjadj et al., 2020).

Consistent with the low circulating levels of IFNs, transcriptional analysis of post-mortem lung samples further confirmed these observations and revealed no detectable type I or Type III IFNs. Among the SARS-CoV-2 proteins which directly interfere with IFN response, ORF6, ORF8, and N protein inhibit IFN-β and NF-κB signaling (Li J.Y. et al., 2020). Further, Konno et al. (2020) have identified a more extended variant of ORF3b with presumably more vigorous anti-IFN activity. Thus, these early observations may point towards an impaired early IFN response by the host cells against SARS-CoV-2

Adding to the essential role of IFN in early antiviral response, two recent studies have shown that genetic changes are associated with inadequate IFN response. In the first study, the presence of IFN neutralizing auto-antibodies found in patients who exhibited more severe disease condition (Bastard et al., 2020). These auto-antibodies were more prevalent in men than women, that partly explains the susceptibility of men to COVID-19. None of the asymptomatic or mild cases had detectable auto-antibodies. In the other study, mutations in 13 key genes implicated in TLR3- and IRF7-dependent exhibit loss-of-function (Zhang Q. et al., 2020). Patients or the cells derived from these patients with loss-of-function in these genes had inadequate IFN response and vulnerable to SARS-CoV-2 infection. In a similar study on four patients with severe disease symptoms, the whole exome-sequencing revealed loss-of-function of TLR7, which is essentially involved in IFN signaling. These patients exhibited decreased expression of IRF7, IFNB1, and ISG15, along with reduced production of IFN-γ (Van Der Made et al., 2020). Thus, impaired IFN signaling, mediated either directly by the virus by interfering at various steps in the IFN signaling, or genetic predisposition of some individuals to inadequate IFN response and presence of IFN neutralizing auto-antibodies are some of the significant factors which determine the COVID-19 disease severity. The dysfunctional IFN response in conjunction with other innate and adaptive immune responses may thus decide the path to recovery or progression to more severe form of the disease (Hadjadj et al., 2020). Impaired type I interferon activity and exacerbated inflammatory responses in severe COVID-19 patients (Hadjadj et al., 2020; Park and Iwasaki, 2020). A comprehensive understanding of the molecular mechanisms by which SARS-CoV-2 causes impaired IFN response is still lacking, and future studies may help us to understand this.

Nevertheless, these initial reports, along with the previous findings on SARS-CoV, are the basis behind exploring the therapeutic efficacy of IFN treatment for COVID-19 patients. Currently, there are ongoing clinical trials with IFN-β1a (NCT04350671), which is in phase II, and IFN-l (NCT04388709) for the treatment of COVID-19. The preliminary results with these drugs have been encouraging as of now (Davoudi-Monfared et al., 2020).

#### Release of Damage-Associated Molecular Patterns and Proinflammatory Molecules

The impaired early IFN response results in high viral propagation that subsequently leads to the induction of a robust proinflammatory response (Davidson et al., 2015). The cytopathic nature of these viruses induces substantial death in infected ATII cells (apoptotic as well as necrotic) which leads to the release of a wide range of damage-associated molecular patterns (DAMPs) and cytotoxic molecules. Similarly, activated AMs also respond to the released DAMPs and act concurrently with PAMPs to amplify the proinflammatory response. A list and role of potential PAMPs, DAMPs, and their respective PRRs have been reviewed previously (Leiva-Juárez et al., 2018).

Circulating nuclear and mitochondrial DNA, and histones serve as potential DAMPs during viral infections. These molecules signal via the TLR pathway and induce robust expression of proinflammatory molecules. Among the DAMPs secreted by virus-infected and damaged epithelial cells, the role of high-mobility group box one protein (HMGB1) and S100 are well known (Leiva-Juárez et al., 2018; Gong et al., 2020). HMGB1 after binding to TLR4 induces activation of NF-κB signaling and release of proinflammatory molecules. Additionally, HMGB1 also activates receptors like TREM1/2, and receptors for advanced glycation end products (RAGE) which are also involved in NF-κB activation (Yang and Tracey, 2010). S100 initiates similar downstream signaling after binding with TLR4 and RAGE receptors (Ma et al., 2017), these studies were recently reviewed by Gong et al. (2020). Previous animal studies with other respiratory viruses have shown a close correlation of increased serum HMGB1 levels with lung injury and disease severity (Patel et al., 2018). Similarly, elevated expression of S100A9 was present in patients during acute lung injury mediated by the respiratory syncytial viral (RSV; Foronjy et al., 2016). Although as of now, presence of HMGB1 has no report in COVID-19 patients, the damage in the lung parenchyma in post-mortem biopsies suggests that it is highly likely that this protein may implicate in disease pathogenesis and hyperinflammation (Andersson et al., 2020; Zhang Q. et al., 2020).

Increased expressions of S100A8, S100A9, and S100A12 calgranulins found in the BALF fluid from COVID-19 patients indicate their potential role in generating the proinflammatory response (Zhou Z. et al., 2020). Further, Zou et al. (2020) showed increased presence of cell-free DNA and citrullinated histones in blood samples obtained from 50 COVID-19 patients. Studies on other inflammatory diseases have shown a close correlation between the presence of these molecules with disease severity (Resman Rus et al., 2016). However, their functional role is yet unexplored, but the increased expression of some of these DAMPs in COVID-19 patients suggests their potential implication in disease pathogenesis. Future studies will clarify the involvement of various other DAMPs in perpetuating the proinflammatory state, and specifically the role of HMGB1.

In addition to the secretion of DAMPs, AM and virus infected ATII cells secrete a range of pro-inflammatory molecules (Hussell and Bell, 2014; Glaser et al., 2019). Among these, increased IL-6 levels are consistently detected in cultured cells infected with SARS-CoV and SARS-CoV-2 (Ye et al., 2018; Herold et al., 2020; Liu J. et al., 2020; Liu T. et al., 2020). Notably, levels of TNF-α, IL-8, IL-10, GM-CSF, CXCL10, and CCL5 secreted by infected ATII and activated AMs were also consistently shown to increase during SARS-CoV and SARS-CoV-2 infections (Ward et al., 2005; Huang C. et al., 2020; Patterson et al., 2020). Transcriptional profiling of cytokines and chemokines in normal human lung epithelial cells (NHBE) infected with SARS-CoV-2 revealed increased levels of CCL20, CXCL1, IL-1B, IL-6, CXCL3, CXCL5, CXCL6, CXCL2, CXCL16, and TNF-α by primary lung epithelial cells in response to SARS-CoV-2 infection (Blanco-Melo et al., 2020). Thus, lung resident ATII and AM cells besides being integral to the antiviral response also participate in generating a profound proinflammatory state.

#### Proinflammatory Molecules Released by Infiltrating Myeloid Cells

##### Circulating inflammatory monocytes/macrophages

A detailed account of the role of inflammatory macrophages in the pathogenesis of SARS-CoV is reported by He et al. (2007). Animal studies have demonstrated extensive recruitment and accumulation of these cells in the lungs, which correlated with the release of TNF-α, IL-1β, and IL-6 and the development of ARDS, reviewed by Gralinski and Baric (2015). Interestingly, depletion of these inflammatory macrophages in animals infected with SARS-CoV was associated with a high recovery rate, thus suggesting their critical role in disease pathogenesis (Channappanavar et al., 2016). Similarly, SARS-CoV infection in animals with STAT1 knockout in alternatively activated macrophages displayed attenuated lung damage and protection from disease (Page et al., 2012). Besides, a large number of clinical studies support an integral role of IMMs in SARS-CoV infected patients (Wong et al., 2004; Tisoncik et al., 2012; Liu et al., 2019). Recent studies from BALF from COVID-19 patients have also demonstrated the critical role of circulating monocyte-derived macrophages in the induction of robust proinflammatory reaction (Liao et al., 2020). Blood cell analysis of 18 COVID-19 patients revealed an activated status of inflammatory macrophages (Zhang D. et al., 2020). In line with these findings, scRNA-seq followed by immune cell profiling of blood cells revealed an increased number of CD14++ monocytes (Wen et al., 2020). Severe and critically ill patients also exhibit macrophage activation syndrome (MAS) in some cases (Giamarellos-Bourboulis et al., 2020). Thus, all the evidence directs towards a critical role of inflammatory macrophages in disease severity during COVID-19 and a potential therapeutic target. Intervention which reduces the impetus to induce MAS like antibodies directed against IL-6 and IL-1β has shown promising clinical outcomes, reviewed by Otsuka and Seino (2020).

##### Proinflammatory neutrophils

Like other innate immune cells, neutrophils are protective in the early phases of infection by neutralizing the viral particles and release of protective molecules to interfere with the viral propagation (Drescher and Bai, 2013). However, in severe cases, the number of these cells increases at the sites of infection and they become the leading damage-causing cells. Excessive infiltration of these cells in the lungs is associated with secretion of TNF-α, IL-6, IL-1β, IL-7, IL-23, and IL-36, along with a broad range of other cytokines and damage-causing neutrophil extracellular traps (NETs; Tecchio et al., 2014). Additionally, these neutrophils also secrete a range of chemokines like CCL2/3/4, CXCL1-13 to attract more neutrophils and monocytes from the circulation (Sokol and Luster, 2015).

Emerging evidence suggests a pivotal role of neutrophils in the pathogenesis of COVID-19. Immune cell profiling revealed activated status of these cells which was associated with increased levels of NETs and correlated with acute-phase reaction (Chen G. et al., 2020; Qin et al., 2020; Zuo et al., 2020). Similarly, an increase in the number of activated neutrophils was present in the BALF of COVID-19 patients (Liao et al., 2020; Xiong et al., 2020). Thus, based on these recently published studies, the neutrophil number in the blood can be used as a predictive marker for disease severity (Zhang et al., 2020a).

##### Natural killer cells

Natural killer cells are essential in the early phase of viral infection to assist in the clearance of the virus by interacting with death receptors expressed on the infected cells (Vidal et al., 2011). Previous clinical studies have shown decreased NK cell number in SARS-CoV patients, which was more pronounced in severe cases (Wang and Xia, 2004). A recent blood profile of COVID-19 patients suggested a similar decline in the number of NK cells in severe cases, along with an increased expression of exhaustion markers (Chen X. et al., 2020; Tan L. et al., 2020b; Zheng H.Y. et al., 2020). On the contrary, no significant difference was found in the number of total NK cells, in non-ICU vs 10 ICU admitted patients (Zhou et al., 2020a). This discrepancy in number could probably be due to differential temporal immune response and the underlying prevailing disease conditions in some patients. Immune cell profiling data from early recovery stage (ERS) and late recovery stage (LRS) COVID-19 patients revealed a biphasic effect, with fewer NK cells during early recovery ERS, which recovered during LRS (Wen et al., 2020). Thus, besides the underlying disease state, the NK cell number may also be sensitive to the time of sample collection and hence may not serve as a potential disease marker. Further, these studies could also suffer from the limitation of the variation in the age of the patients studied which may make it difficult to provide a definite role of these cells concerning COVID-19 disease severity (Nikolich-Zugich et al., 2020), necessitating more conclusive studies.

##### Lung resident and monocyte-derived dendritic cells

Lung resident dendritic cells majorly have a protective role during the early onset of the disease by activating the adaptive immune cell response. Under the influence of PAMPs, DAMPs, and inflammatory cytokine signaling, lung resident dendritic cells are conditioned and migrate to the draining lymph node under the influence of CCR7 where they prime naïve CD4+ and CD8+ T cells (Braun et al., 2011; Thaiss et al., 2011). In contrast, monocyte-derived dendritic cells generate under the influence of GM-CSF, IFN-γ, and IL-4, along with other proinflammatory signals (Qu et al., 2014). Previous studies have shown elevated secretions of CCL3, CCL5, MCP-1, IP-10, TNF-α, and IL-6 by activated inflammatory dendritic cells (DCs) in response to SARS-CoV (Law et al., 2005). Recent reports also suggest the presence of activated dendritic cells in COVID-19 patients. Notably, meta-transcriptomic sequencing of BALF obtained from 8 COVID-19 patients revealed an activated status of these cells along with neutrophils, as compared to other innate and adaptive immune cells (Yang A.P. et al., 2020; Zhou Z. et al., 2020). Thus, based on previous clinical studies on SARS-CoV infection and recent emerging studies on SARS-CoV-2, it is evident that hyperinflammatory immune response in severe and critically ill COVID-19 patients is mainly mounted by infiltrated innate immune cells at the site of infection with a substantial contribution by the adaptive immune cells as discussed below in the section on the dysfunctional adaptive immune response.

## Adaptive Immune Response in COVID-19

### Functional Adaptive Immune Response

The functional but well-regulated adaptive immune response is necessary to overcome the viral infection. Specifically, T cells when recruited to the site of infection engage in eliminating the infected cells and act in concordance with virus-specific neutralization antibodies to provide sustained immunity (Hor et al., 2015; De Biasi et al., 2020). Considering the recent extensive work in understanding the functional early immune response during COVID-19, it appears that a complex interplay between T and B cell immune response along with patient-specific underlying health condition and genetic factors determines the recovery, as will be discussed in following sections.

#### T Cell Response

Generation of early adaptive immune response is critical for the selective elimination of virus-infected cells and neutralization of viral antigens, thereby preventing the damage to the underlying lung parenchyma. Cytokines, chemokines, PAMPs, and DAMPs released by infected ATII and activated AMs in the lung are adequate to mount a well-coordinated and regulated adaptive immune response by priming lung resident DCs. After encountering the antigen-presenting DCs, naive CD4+ T cells differentiate into effector and memory CD4+ T cells. At least five different CD4+ T cell lineages are known (TH1, TH2, TH17, TFH, and TREG cells) with prominent roles of TH1 and TFH cells in mounting antiviral response during SARS-CoV infection (Channappanavar et al., 2014). Additionally, some studies have also shown a functional TH2 response in PBMCs derived from COVID-19 patients. Release of TH2 specific cytokines like IL-4 and IL-5 was observed in vitro after these cells were stimulated (Weiskopf et al., 2020). Similarly, these patients show enhanced production of IL-17 along with other TH17 cell-specific cytokines (Liu J. et al., 2020; Wu and Yang, 2020). These findings suggest that the TH cell response in COVID-19 patients is complex concerning other infections, and this complexity may partly depend upon the prevailing pathophysiological state of a patient.

During viral infections like SARS-CoV, TH1 differentiation is influenced by IL-12 and IFN-γ secreted by DCs along with co-stimulatory signaling via B7-1/2 and CD28. Whereas IL-6 secreted by DCs influence TFH differentiation to aid in antibody secretion by B cells (Tang et al., 2008; Lau et al., 2012). Under the influence of chemokines (CCL3, CCL4, CCL5, CCL8), TH1 cells are recruited to the site of infection and are distinguished by the secretion of IL-2, IFN-γ, IL-12, and TNF-α as the main effector cytokines during SARS-CoV infections (Li et al., 2008). Similarly, naive CD8+ T cells are activated by DCs by engaging MHC-I and TCR receptors, along with CD28-B7 co-stimulatory signaling and cytokines released by CD4+ T cells. IL-2 secreted chiefly by CD4+ T cells is also implicated in their long-term maintenance and proliferation (Eickhoff et al., 2015; Hor et al., 2015). Notably, CD8+ T cells could also be activated independently of help from CD4+ T cells under conditions where a robust IFN Type I response is present (Wiesel and Oxenius, 2012). These activated CD8+ T cells [also referred to as cytotoxic T lymphocytes (CTLs)] get subsequently recruited to the effector organ under the influence of chemokines (CCL3, CCL4, CCL5, CXCL9, and CXCL10) (Nolz, 2015). At the infected site, CTLs mount an antiviral response by directly killing the infected cells via secretion of cytotoxic molecules like granzymes, perforins, granulysin, and other cytotoxic granules. Very recently, a study shows that CTLs secrete the granzymes and perforins as supramolecular attack particles (SMAPs) in a glycoprotein complex along with over 283 other proteins (including cytokines such as IFN-γ and TNF-α) (Bálint et al., 2020). It will be interesting to know whether infection by CoVs also influences the release of SMAP by CTLs.

Animal studies have revealed the critical molecular insights of CD4+ cells in SARS-CoV clearance and attenuation of a pathological condition. The depletion of CD4+ cells was associated with reduced virus clearance and interstitial pneumonitis (Jin et al., 2005; Wang et al., 2006). In comparison, the adoptive transfer of virus-specific CD4+ and CD8+ T cells resulted in viral clearance (Zhao et al., 2010). Similarly, clinical data has consistently shown the presence of antigen-specific CD4+ and CD8+ T cells in the recovered patients, akin to what was found in immunized animals, reviewed in Channappanavar et al. (2014). On the other hand, severe cases of SARS-CoV infection were associated with a decline in T cells, as will be discussed in later sections. Thus, based on these animal and clinical data, CD4+ T and CD8+ T cells were central to the antiviral response during SARS-CoV infection (Peng et al., 2006; Oh et al., 2012).

A subset of primed CD4+ and CD8+ T cells differentiates into long-acting memory cells after the infection subsides. TCR-p: MHCII signaling helps in CD4+ T memory cell formation along with presence of cytokines like IL-2, IL-21 and interaction via CD40R-CD40L (Jaigirdar and MacLeod, 2015). Similarly, This CD8+ T cell transition to memory cells take place under the influence of CD8+ TREG cells via secreted IL-10 (Laidlaw et al., 2015). Long lasting CD4+ and CD8+ T memory cells were detected in the recovered SARS-CoV infected patients (Peng et al., 2006; Li et al., 2008).

Besides, other T cells subsets which are involved in antiviral response include unconventional NKT cells (CD56+) and MAIT (mucosa-associated invariant T) cells. NKT cells act at the interface between innate and adaptive immune response and traffic to the site of infection under the influence of cytokines (Tsay and Zouali, 2018). MAIT cells reside in the mucosal lining, such as in the lungs where they serve an immunoregulatory function. Both these cell types play an essential role in the early clearance of the SARS-CoV-2, along with other T cell subsets (Grifoni et al., 2020). Strategies to enhance their function are proposed to enhance the virial clearance during COVID-19 (Cao, 2020). Role of these cells will be further discussed under the dysfunctional immune response in section “T and B Cell Response in Mild/Moderate and Recovered COVID-19 Patients.”

#### B Cell Response

B cells, along with T cells, form the central adaptive response during viral infections. B cell response is highly specific, mounted by the virus-specific antibodies and other effector cytokines secreted by these cells. B cell activation can be follicular helper T (TFH) cell-dependent, or in some instances, independent of helper cells; both instances are prevalent in COVID-19 (Mathew et al., 2020). Under the influence of antigen-presenting dendritic cells, naïve CD4+ T cells differentiate into TFH cells, which are marked by high expressions of CXCR5 and IL-21, and low expressions of CCR7, IFN-γ, IL-4, and IL-17 (Rasheed et al., 2006; Nurieva et al., 2008; Morita et al., 2011). The activated TFH cells interact with B cells via CD40R-CD40L and other associated receptors to induce the production of antigen-specific antibodies in a well-coordinated and regulated process. This CD40R-CD40L interaction along with the secretion of IL-21 also allows the formation of long-lived memory B cells, while B cell-derived IL-6 and IL-27 help in reciprocal maintenance of TFH cells (Nurieva et al., 2008, 2009). A previous animal study has shown the essential role of these helper cells in mounting an adequate antibody response against SARS-CoV infection (Chen et al., 2010). The depletion of these cells was associated with a decline in antibody response and reduced viral clearance. Thus, virus-specific antibodies produced by B cells are critical for an effective immune response mounted by the host. These antibodies facilitate the clearance of the virus by either directly activating phagocytosis, opsonization, or activation of the antibody-dependent cellular cytotoxicity (ADCC) via effector NK cells. Cytokines released by the activation of innate and adaptive immune systems also activate the complement system. Viruses coated with the secreted antibodies from plasma cells eventually get eliminated by the complement system, reviewed by Risitano et al. (2020).

#### T and B Cell Response in Mild/Moderate and Recovered COVID-19 Patients

T cell response is an emerging critical determinant in keeping the SARS-CoV-2 infection under check (Huang C. et al., 2020; Liu J. et al., 2020). Across studies, a decline in the number of these cells positively correlates with poor clinical outcome and immuno-pathogenesis, whereas adequate T cell number and proper effector function are prevalent in patients who develop mild disease symptoms or those who successfully recovered (Chen G. et al., 2020; Li H. et al., 2020; Sekine et al., 2020; Tan L. et al., 2020b). Following a single patient (47-year-old woman) throughout the disease, Thevarajan et al. (2020) showed a concomitant increase in CD4+, CD8+, TFH cells, and antibody-secreting B cells from day seven after infection, which persisted for a week as the symptoms resolved. Other studies revealed a similar trend of revival in T cell response in patients who have successfully cleared the virus (Anft et al., 2020; Braun et al., 2020; Chen X. et al., 2020; Chen N. et al., 2020).

SARS-CoV-2 specific reactive CD4+ and CD8+ T cells were found in 100 and 80% patients who needed mechanical ventilation (*n* = 10). PBMCs derived from these patients showed reactivity against the S protein of SARS-CoV-2. Further, in vitro stimulation of CD4+ T cells led to their differentiation into TH1, TH2, and TH17 subsets, as revealed by the expression of their corresponding cytokines (Weiskopf et al., 2020). Interestingly, 20% of non-infected healthy controls also displayed reactive T cells. The main limitation with this study was that the T response was studied only in critically ill patients and the small sample size was small to provide.

By studying a cohort of 18 COVID-19 patients and 64 healthy donors, Braun et al. (2020) found reactive CD4+ (83%) cells in blood-derived from the convalescing COVID-19 patients. These reactive T cells were found specifically against the S protein. Interestingly about 35% of SARS-CoV-2 seronegative healthy donors also showed the presence of S protein reactive CD4+ T cells indicating previous exposure to the related coronavirus infections. Simultaneously, another study has found SARS-CoV-2 specific CD4+ T (100%) and CD8+ T (70%) cells in convalescent patients (*n* = 20) (Grifoni et al., 2020). In addition to being majorly reactive against S protein, the study found additional targets of these T cells in the form of M, N, and ORF8 proteins and other non-structural proteins like NSP3, NSP4, ORF3a. Further, in line with the study by Braun et al. (2020), T cells were found reactive against 40–60% of the SARS-CoV-2 uninfected patients, suggesting the presence of these reactive cells in response to previous viral infections.

In a yet to be a peer-reviewed article, Schulien et al. (2020) has extensively studied the SARS-CoV-2 epitope-specific role of CD8+ T cells in COVID-19 (Schulien et al., 2020). The study found the presence of newly generated and pre-existing SARS-CoV-2 specific cells with the positive response seen in 88.4% of patients who had mild disease symptoms (*n* = 26). The most substantial response was found against N protein and ORF3a. Further, CD8+ T cells response was shown persistent even in the individuals who became seronegative. In a patient studied longitudinally (70 days), CD8+ T cell response prolonged but antibody did not persist. All these three studies taken together point toward the presence of functional and long-lasting reactive T cells in convalescent individuals, while others also suggest the presence of reactive T cells in critically ill patients (Weiskopf et al., 2020). Thus, based on these studies, it appears that COVID-19 patients who exhibit mild disease symptoms and successfully recover, display functional and long-lasting T cell response. However, these findings may not be definitive to provide a coherent functional view of these cells during recovery, as none of these studies compared the T cell response to disease severity. A further difference in the time of sample collection may also complicate the findings. In the study by Grifoni et al. (2020) samples were collected throughout 20–35 days after symptom onset, whereas Weiskopf et al. (2020), used samples collected after 14 days of ICU admission. Thus, more studies under controlled clinical settings and large cohort size are warranted.

While addressing some of these concerns, a recent study explored T cell response in convalescent COVID-19 patients concerning disease severity (Peng et al., 2020). The study found robust CD4+ and CD8+ memory T cell response in severe cases (*n* = 14) than mild (*n* = 28), suggesting long-lasting memory of these cells to keep the infection in check. The limitation again here is the small sample size. Therefore, more such studies with large sample size are needed to fully understand the impact of T cell response and its long-term sustainability.

B cell response has a temporal dynamic to human infecting CoVs, with a median time of detection for SARS-CoV as 14 days, reviewed by Huang A.T. et al. (2020). The peak antibody titer for IgG and IgM, and detection time of neutralizing antibody varied across studies with a lower time point of seroconversion for IgG, IgM, and IgA as 15 days (Hsueh et al., 2004; Mo et al., 2006; Cao et al., 2007; Yang et al., 2009). A more dynamic range of seroconversion was observed in sera from the COVID-19 patients. A study by Liu X. et al. (2020) on 32 patients with varying disease severity has shown detectable IgM antibodies from day four and peaked at day 20, since the onset of the symptoms. At the same time, IgG antibodies appeared after day 7 with a peak on day 25. When compared to the disease severity, mild cases had peak IgM response earlier than in severe cases (day 17 vs day 21). Further, severe cases exhibited more robust IgG antibody response than mild cases, as will be discussed in the subsequent section C. In terms of the antibody response seen after symptom onset, a similar trend was shown by Liu X. et al. (2020) who detected IgM antibodies in SARS-CoV-2 infected patients between 3 and 6 days and IgG antibodies after day 8 of symptom onset, irrespective of the disease severity.

**FIGURE 3:**
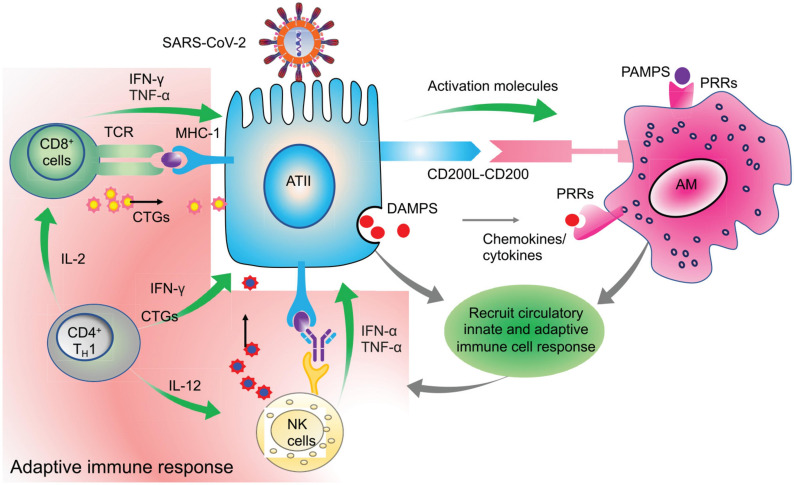
Clearance of virus infected cells by engaging adaptive immune cells. Virus infected ATII cells activate the neighboring lung resident AMs by minimizing the CD200-200L interaction. Additional requisite activation signals are provided by DAMPs, viral derived PAMPs, and cytokines like IFN-γ. Activated AMs along with infected ATII derived molecules activate and recruit other innate immune cells, like circulating monocytes, dendritic cells, NK cells, and neutrophils which act in a coordinated manner to eventually recruit the adaptive effector immune cells like CTLs and CD4^+^T cells. These adaptive immune cells then specifically eliminate virus infected cells while minimizing the damage to the nearby uninfected cells. Thus, a well-coordinated and regulated adaptive immune response with help from innate immune cells is critical for initial antiviral response to limit the further spread of the virus. Green arrows indicate the cytokines released by the respective activated immune cells which activate other immune cells as well as mount an antiviral response by acting on lung epithelial cells.

A study by Zhou P. et al. (2020) also found mean times of IgM, IgG, and neutralizing antibodies at 12, 14, and 11 days, respectively. These reports were consistent with the reports from Wu et al. (2020) in which neutralizing antibodies were detected starting from day 10. An elaborate antibody profile of 285 COVID-19 patients revealed 100% IgG and 94.1% IgM antibody response with a peak around the 3rd and 4th week after symptom onset, respectively (Long et al., 2020a). Thus, for a successful viral clearance, an adequate adaptive immune response is generated around 2nd week after symptom onset and peaks around the 3rd week for IgM and at the beginning of 4th week for IgG (Ni et al., 2020; Thevarajan et al., 2020; Wu et al., 2020; Zhao et al., 2020). Based on these and several other studies, it is evident that the antibody response is very dynamic in COVID-19 which may be dependent on the age, sex, genetic factors, underlying disease condition and most importantly, the type of assay used for serological testing (Guan et al., 2020; Hou et al., 2020). Overall, these initial reports unequivocally suggest an integral role of the regulated adaptive immune response in the early clearance of virus and thereby attenuation of the disease condition in almost 80% of the patients who show mild/moderate symptoms. On the other hand, in the rest, 20% severe and critically ill patients, disease symptoms positively correlate with the degree of lymphocytopenia, as will be discussed later in section C. A schematic representation of the functional immune response during COVID-19 is depicted in Figure 3.

An immunological enigma still eluding researchers worldwide is how the majority of COVID-19 patients remain asymptomatic, and even some with high viral load (Lee S. et al., 2020). This dilemma can be partly explained based on the effective functional early immune response generated by the T and B cells. Mathew et al. (2020) used a multidimensional immunoprobing study and functionally characterized clinical features with immunological features. This study defined three immunotypes based on 50 clinical and 200 immune parameters. The immunotype 1 was positively associated with disease severity and had hyperactivated CD4+ and CD8+ T cells, with concomitant expression of exhaustion markers, indicating robust activation followed by the exhaustion of these cells. This immunotype may thus be vulnerable to cytokine storm, as discussed later in section “Cytokine Storm in COVID-19 Patients.” Immunotype 2 was associated with the presence of proliferating memory B cells with the optimal activation status of CD4+ and CD8+ T cells. This immunotype did not associate with disease severity. The immunotype 3 had no activation status of CD4+ and CD8+ T cells, and thus exhibited an inverse correlation with the disease severity. Overall, this study addressed some of the above questions that suggested that the presence of a regulated and functional adaptive immune response is key to preventing immunopathology. In a similar study, the activation status of T cells associated with disease severity (acute, moderate, and severe) (Sekine et al., 2020). The activation status of these T cells correlated with the presence of SARS-CoV-2 specific IgG antibodies in these patients.

Interestingly, T cells derived from convalescent mild and asymptomatic patients exhibited functional status when stimulated in vitro with SARS-CoV-2 specific antigens, suggesting the presence of well-regulated and functional T cell response in mild and asymptomatic convalescent patients. Thus, in patients with high viral load, an immunopathological state can be prevented if the adequate and regulated adaptive immune response is present in association with the proper interferon response. While in patients with compromised immune response, like in comorbid conditions, even a low viral load is sufficient to induce immunopathological changes, due to either ineffective immune response or uncontrolled hyper-activated response, as will be discussed in the subsequent sections.

### Dysfunctional Adaptive Immune Response

A subset of COVID-19 patients displays robust activation of T and B cells. These exaggerated T cell responses are specifically present in patients who manifest severe disease conditions and need mechanical ventilation (Herold et al., 2020). Further, analysis of peripheral blood, BALF, and post-mortem lung samples of deceased patients reveal robust activation of T and B cells with a concomitant decline in the number of these cells (Kaneko et al., 2020; Liao et al., 2020). Thus, it is becoming apparent that a subset of COVID-19 patients displays activated adaptive immune response, which augments hyper-inflammation, thereby leading to disease worsening. In the subsequent section, we will specifically discuss the intricate role of T and B cells concerning their contribution to the development of the immunopathological state and how this critical antiviral immune response becomes awry during COVID-19.

#### Proinflammatory Cytokines Secreted by T Cells During COVID-19

Hyperinflammatory condition mediated by cytokines, chemokines and associated proinflammatory molecules which are secreted by both innate and adaptive immune cells. However, during COVID-19, the relative contribution of adaptive immune cells towards proinflammatory molecules is still emerging, while the published studies suggest a complex interplay. Profiling of 21 cytokines and chemokines in 39 patients and 24 healthy controls revealed increased levels of TH1 specific cytokines like IFN-γ, IL-2, and IL-12, and TH17 specific IL-17 in peripheral blood. In comparison to the mild cases (*n* = 19), patients with severe disease (*n* = 10) condition had increased levels of these cytokines. The limitation of this study was that the median age of severe cases was higher than in mild cases (Song et al., 2020).

Similarly, Zhou et al. (2020b) reported hyperactivated TH1 cell response with increased secretion of IFN-γ, GM-CSF, and IL-6 and with more robust expression in ICU cases than non-ICU. Considering the age, gender and other associated factors, a large number of other studies have now confirmed that COVID-19 patients have increased levels of TH1 specific cytokines, with more robust levels seen in severe than mild cases (Huang C. et al., 2020; Xu Z. et al., 2020; Zhou et al., 2020b). Similarly, CD8+ T cell-specific cytokines increased in COVID-19 patients, more pronounced in severe than mild condition (Zhou et al., 2020b). Increased expression of GM-CSF was found in CD8+ T cells from ICU patients than non-ICU, while no difference was observed in IL-6 and TNF-α levels. PBMCs derived from COVID-19 patients and stimulated in vitro showed an increase in expression of CCL2, CXCL10, Eotaxin, and IL-1RA, and stimulation of CD8+ T cells were associated with an increase in IFN-γ levels, which indicates the functional responsiveness of these cells (Mathew et al., 2020). These studies thus suggest a robust activation of TH1 specific and CD8+ T cells in COVID-19 patients.

On the contrary, there are studies which show decreased cytokine expression by T cells in severe COVID-19 cases. A study by Zheng H.Y. et al. (2020) showed a lower expression of IFN-γ, IL-2, and TNF-α in CD4+ T cells derived from severe cases. Similarly, a decrease in IL-2+ CD8+ and IFN-γ+ CD8+ cells was also observed (Diao et al., 2020). Although most studies point toward the robust activation and release of proinflammatory cytokines by CD4+ and CD8+ T cells, the discrepancy in latter studies could attribute to the functional exhaustion of these cells, which will we will discuss in section “Lymphocytopenia During COVID-19.”

Besides the presence of TH1 cytokines, TH2 cytokines like IL-4 and IL-5 and TH17 specific IL-17 were reported in some studies (Han et al., 2020; Huang C. et al., 2020; Song et al., 2020; Tan L. et al., 2020b; Xu Z. et al., 2020). The presence of TH2 cytokines usually seen in mild cases may be accounted for by the presence of other respiratory conditions with TH2 specific response (Laing et al., 2020). Overall, all these studies point toward the increased secretion of proinflammatory molecules by T lymphocytes in COVID-19, albeit with a heterogeneous response, which may be due to the variation in the age of the patients studied, different sampling times and presence of the comorbid condition, which needs further investigation.

#### Activation and Exhaustion Status of T Cells During COVID-19 Infection

The activation, exhaustion, and proliferation response of T and B cells are considered an integral determinant of the disease severity. Unequivocally, studies have shown lymphocytopenia as a predictive marker which may also determine the disease severity in COVID-19 patients (Liu J. et al., 2020; Tan L. et al., 2020b; Wang et al., 2020b; Yang A.P. et al., 2020; Yang X. et al., 2020; Zhang et al., 2020a). However, contradictory reports exist regarding the functional and exhaustion status of these cells during COVID-19. Further, understanding these changes throughout the disease has remained a challenge, considering the complexity in the underlying immune response, comorbid condition, and previous exposure to the infections.

Peripheral blood study of a single patient (50-year male) revealed robust activation of CD4+ and CD8+ T cells marked by HLA-DR expression (Xu Z. et al., 2020). However, the major limitation of this study was that only a single patient was studied. Using multiparameter flow cytometry approach Kuri-Cervantes et al. (2020) studied 35 COVID-19 patients (*n* = 7 moderate and *n* = 28 severe). The study revealed that a subset of severe cases displayed T cell activation as revealed by CD38 and HLA-DR expression in both CD4+ and CD8+ T cells (Kuri-Cervantes et al., 2020). By analyzing, PBMCs derived from healthy (*n* = 5) and severe cases (*n* = 16), the authors found an increase in the percentage of cytotoxic CD8+ memory cells as revealed by perforin and granzyme B.

Similarly, a subset of severe cases had increased Ki-67 expressing CD4+ and CD8+ T cells, displaying proliferation. At the same time, these findings revealed heterogeneous T cell response but overall suggested a skew towards the activation and proliferation status of these cells in a subset of severe cases. The limitation of this finding is again the small sample size which may be the reason for the inconclusive findings of the T cell status concerning the disease severity.

Similar multiparameter flow cytometry approach was used by De Biasi et al. (2020) to study T cell response in healthy (*n* = 12) and COVID-19 patients (*n* = 21). The study found activated status of CD4+ and CD8+ T cells as revealed by an increase in CD38+HLA-D population. Activated status of the CD4+ T and CD8+ T cells was further confirmed by production of IFN-γ, TNF-α, IL-17, and IL-2 when stimulated *in vitro*. The major limitation of this study was that the sample size was small, which restricted the comparison between the T cell responses across patients with various disease severity. In another study, Song et al. (2020) showed the activated status of CD8+ T but not CD4+ T cells in severe (*n* = 9) than mild (*n* = 20) patients. The activated status of CD8+ T cells reflected by the increased population of CD38+HLA-DR+, HLA-DR+, and CD38+HLA-DR+ marker expression (Song et al., 2020). Further, CD8+ T cells were associated with increased cytolytic markers like granzyme B, perforin, and granulysin with more pronounced activation in severe than mild.

While across studies, it has become apparent that T cells show robust activation status in severe cases than mild and moderate. These cells also exhibit exhaustion status, which may occur concomitantly with their activation status. Deep immune profiling of 125 patients by Mathew et al. (2020) demonstrated that both CD4+ and CD8+ T cells exhibit activation status as revealed by coexpression of CD38 and HLA-DR which corresponded to the disease severity. Further, these cells were also associated with concomitant expression of proliferation (Ki-67) and exhaustion (PD-1) markers. This study thus suggests that hyperactivated status of T cells may eventually lead to their exhaustion, and thus these functional and exhaustion features of T cells may reflect the disease severity.

A study by Zheng M. et al. (2020) in a cohort of 68 COVID-19 patients revealed extensive CD8+ T cell exhaustion as shown by increased expression of NKG2A. Intracellular cytokine staining (IFN-γ, IL-2, and granzyme B) further confirmed a decrease in the activation profile of these cells, which was more pronounced in severe (*n* = 55) than mild (*n* = 13) cases (Zheng M. et al., 2020). As mentioned earlier in the study by Song et al. (2020) and De Biasi et al. (2020) T cells showed activation status that was also concomitantly seen with express of exhaustion markers PD-1 and TIM-3 on CD8+ T cells and TIM-3 on CD4+ cells. The exhaustion was more pronounced in severe cases (*n* = 9) than mild (*n* = 20). However, both these studies did not consider the age of the patients when comparing the disease severity. Further, the study did not consider the temporal dynamics of these cells while measuring their functional properties.

In agreement, Zheng H.Y. et al. (2020) showed reduced functional activation of CD4+ T cells in severe (*n* = 6) than mild (*n* = 10) group as revealed by a lower proportion of IFN-γ and IL-2 expressing CD4+ T cells. While IL-2 expressing CD4+ T cell population was also significantly lower in healthy vs mild group. Further, CD8+ T cells displayed exhaustion as revealed by an increase in CTLA-4 in severe cases than mild and TGIT in severe than healthy, while PD-1 was more in mild than healthy. Exhaustive states of both CD4+ and CD8+ T cells were also present in patients requiring ICU (Diao et al., 2020). The exhaustive state was apparent by an increase in PD-1 and Tim-3 expression, which was more pronounced in CD8+ than CD4+ T cells. These studies along with others thus suggest that robust activation followed by the exhaustion of CD4+ and CD8+ T cells may be responsible for the disease progression, while therapies like checkpoint inhibitors (anti-PD-1 antibody; NCT04268537) which may prevent T cell exhaustion and restore their functional state may benefit some patients. More studies are necessary before using such an approach can be used for therapeutic intervention.

**FIGURE 4:**
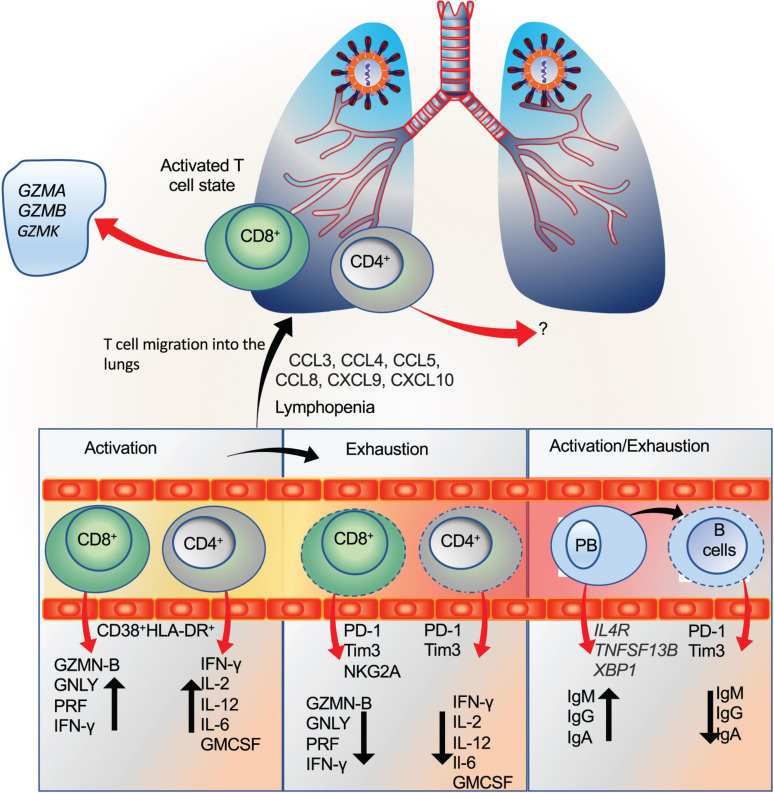
T and B cell immune response during SARS-CoV-2 infection. **(A)** The activation status of CD4^+^ and CD8^+^ T in the circulation is indicated by CD38^+^ HLA-DR^+^. These activated T cells are further recruited at the sites of infection (initially lungs) in the presence of their respective chemokines. The activated CD4^+^ T cells are marked by the presence of cytokines like IFN-γ, IL-2, IL-12, IL-6, and GM-CSF, whereas activated CD8^+^T (cytotoxic T cells) are marked by the secretion of granzymes, perforins, and IFN-γ. During SARS-CoV-2 infection, activated CD8^+^T cells exhibiting increased expression of granzyme A, B, and K (GZM-B, GZM-A, and GZM-K) were found in the lungs (Liao et al., 2020; Song et al., 2020; Zheng M. et al., 2020). **(B)** T cells were also found to exhibit exhausted state as marked by the expression of PD-1, Tim3, and NKG2A. However, most studies showing exhausted T cells were confined to the peripheral blood, while lungs were mostly shown to have activated T cells but with concomitant expression of some exhaustive markers, suggesting that the activation state is followed by exhaustion. The exhaustive T cells are marked by the reduced expression of respective chemokines and cytolytic granules. **(C)** Similarly, antibody-producing B cells (plasmablasts; PB) were shown to exhibit activation status as reflected by the expression of IL4R, TNFSF13B, and XBP1, while at the same time, the exhausted status of these cells was also reported in the peripheral blood. Exhaustive state of B cells is reflected by a decrease in antibody production.

A post-mortem study of deceased COVID-19 patients conducted to find the status of these cells at the site of infection. T cell profiling and their activation status in the lungs revealed an increase in the presence of CD4+ and CD8+ T cells exhibiting activation status (Song et al., 2020). This increase in infiltration of these cells was concomitantly associated with their decline in peripheral blood. Others presented a similar activation profile of CD8+ T cells (Kuri-Cervantes et al., 2020; Mathew et al., 2020). This activated state of CD8+ T cells was consistently present across studies, with reports of immune profiling in BALF samples from COVID-19 patients, which showed increased CD4+ and CD8+ T cells in the lungs in both mild and severe cases along with the increased expression of CD8+ T cell cytolytic genes like GZMA and GZMK (Liao et al., 2020). Thus, these studies point towards heterogeneous activation and exhaustion status of T cells in peripheral blood, while a more consistent activated status at the site of infection (lungs) (Figure 4).

Further, it appears that unlike CD4+ T cells, the activation status of CD8+ T cells is more pronounced, which may account for their relatively faster exhaustion state (Wherry et al., 2007). Interestingly, by studying the CD8+T cell response in convalescent patients, Habel et al. (2020) found that these cells skewed toward naïve, stem cell and central memory phenotypes, with low effector T cells. While comparing the response with Influenza A viruses, SARS-CoV-2 directed CD8+ T exhibit relatively lower response. Others have also shown a significant decline in CD8+ T cell subsets (naïve, effector, and memory) in COVID-19 patients, with a more pronounced decline in critical (*n* = 3) than severe (*n* = 5), and mild (*n* = 4), suggesting their robust activation during early disease followed by exhaustion during the critical condition (Wang W. et al., 2020). On the contrary, CD4+ T cells were higher in the mild and critical cases than severe cases and healthy control (*n* = 12). These results imply that the overall T cell response is heterogenous, while CD8+ response, though robust during infection and correlates with the disease severity; but the response may not be long-lasting, at least in some cases.

Both CD4+ and CD8+ T cells also exhibit dysregulated response (Qin et al., 2020). Decreased levels of CD4+ regulatory cells as marked by CD3+ CD4+ CD25+ CD127low+ population was found in severe cases. Similarly, the study found decreased CD8+ suppressor T cells (CD3+, CD8+, CD28+) in severe cases. Overall, more comprehensive studies are warranted with larger cohort size, to profile local vs systemic T cell response and persistence simultaneously, and correlate these responses with disease severity in age-matched patients.

#### Impaired B Cell Response During COVID-19

Regulated and controlled B cell response is critical for the effective immune response against the CoVs, as discussed above. However, under certain conditions, B cell response may be detrimental and aggravate the underlying disease condition. A notion has emerged, which suggests that in COVID-19 patients, B cell number though reduced, but these cells display robust activation in some cases that correlate with disease severity.

Deep immune profiling integrated with computational approach revealed intricate relations of B cell response with clinical parameters at various stages of the COVID-19 disease severity. These cells express proliferation (Ki67+), differentiation (CD27+ CD38+), as well as exhaustion markers (PD-1+). More robust expression of these markers was observed in severe cases compared to mild-moderate, with an overall decrease in memory B cell number (Mathew et al., 2020). Further, 70% of the patients reported have IgG and IgM S protein-specific antibodies, suggesting activation status of the antibody-secreting plasmablasts. Thus, this study shows that B cells, in severe cases, display concomitant activation and exhaustion markers, while mild cases or healthy controls showed a normal response. However, how this activated status of B cells had an impact on disease severity was not studied. By looking at the alleged relationship of activated B cells with disease severity, Woodruff et al. (2020) showed robust activation status of extrafollicular B cells which resembled their behavior in autoimmune condition. The activation status of these cells was found more pronounced in critically ill patients (*n* = 10) than non-critical (*n* = 7) and healthy control (*n* = 17), which correlated with SARS-CoV-2-specific antibody production and disease progression. Further, an increase in antibody-secreting cells (ASCs) was found in critically ill cases compared to non-severe cases along with an increase in S protein-specific antibodies, probably with a non-neutralizing property. This study shows that in some patients with a critical disease condition, robust B cell response and presence of SARS-CoV-2 antigen-specific antibodies may be associated with worsening of the disease condition. The ASCs were identified as the population of cells with CD138+ and CD21low expression. However, no comparison was drawn between various age groups concerning disease severity. While across studies, B cell activation is apparent in severe cases, it is subsequently associated with a sharp decline in their number. Various mechanisms may be responsible for this decline, among which B cell exhaustion is one, but still poorly understood (Yi et al., 2010).

A recent study has provided mechanistic insights into how some cases of COVID-19 exhibit low B cell number. Kaneko et al. (2020) studied the post-mortem samples (*n* = 11) of thoracic lymph nodes and spleens and found that Bcl-6+ germinal center (GC) B cells highly reduced in these patients in comparison to non-COVID-19 control (*n* = 6). This decline in GC was also associated with a decrease in TFH cell differentiation and an increase in the number of TH1 cells (Kaneko et al., 2020). Further, an increase in expression of TNF-α levels was found in the follicles. Based on previous studies that TNF-α inhibits the lymphoid follicular development, and high levels of this pleiotropic cytokine is the hallmark of COVID-19, the authors attributed the reduction in GC to high levels of this cytokine. In addition to the study in post-mortem samples, the authors conducted B cell analysis in peripheral blood samples from COVID-19 patients at different stages of the disease. In line with the post-mortem data, patients with severe disease condition (*n* = 25) had a significant decrease in the number of naïve B cells, CD19+ B cells, and follicular B cell subsets in comparison to the healthy controls (*n* = 4), convalescent patients (*n* = 39), and moderate patients (*n* = 4). Thus, this study provides a probable cause for the B cell decline in severe cases of Covid-19. However, there was a significant difference in the mean age of severe patients (higher between 58 and 60) than the control, convalescent, and moderate group (30–45 years). Thus, the effect of age on the decline in B cells cannot be undermined in this study. More studies are needed to specifically look into the B cell number and activation status in COVID-19 patients concerning the disease severity to get a clear understanding of the role of these cells.

#### Antibody Dynamics in COVID-19

Antibody-based therapy is being considered as a potential intervention for COVID-19, owing to the successful preliminary results with CPT. However, this treatment approach may be associated with the risk of exacerbating COVID-19 severity, based on the experience from previous viral infections (Salazar et al., 2017). Further, like previous SARS-CoV infections, antibody response may not always favor viral clearance, instead of contributing to the underlying immunopathology in some instances (Zhang et al., 2006; Newton et al., 2016). This immunopathological state may thus attribute to factors such as robust and unregulated activation of B cells, ADE, presence of cross-reactive but non-neutralizing antibodies, and failure to mount a controlled B cell response. Across studies, higher antibody titers detected in patients with severe and critical condition in comparison to non-severe cases (Long et al., 2020a; Gudbjartsson et al., 2020; Zhao et al., 2020). One can argue that higher antibody titer should be beneficial to provide an adequate antiviral response but can be countered by the finding that higher antibody titers found in a large number of severe cases and patients requiring ventilation (Kaneko et al., 2020). This contradiction is yet to resolve, and the emerging data suggest that higher antibody response may reflect the over-activation and uncontrolled B cell response. Zheng M. et al. (2020) showed the presence of RBD-specific IgG and IgA antibodies in patients with severe disease condition. The study included 13 severe and 41 non-severe cases of various age groups.

Along with increased IgG and IgA levels, severe cases also had an increased number of antibody-secreting cells and TFH cells, which aid in antibody production. Further, a close correlation of proinflammatory cytokines and chemokines like IL-6, CXCL10 and complement activation marker C5a found with the severe disease condition. This study provided a direct relation of inflammatory response with humoral immune response in context to the disease severity. However, the antigen-neutralizing property of these SARS-CoV-2 specific antibodies was not determined. Further, a low sample size of severe cases was another limiting factor to provide a definitive conclusion that robust antibody response may positively correlate with disease severity.

Similarly, Zhao et al. (2020) studied antibody response in 173 clinically diagnosed COVID-19 patients with a median age of 48 years. Among these, nine patients (three critical and six non-critical) studied longitudinally for the relation of antibody response with the disease severity. Antibody titer was higher in the critical patients as compared to non-critical. This higher titer of antibodies was not reflected by the clearance of the virus, thus suggesting that antibody response in critical cases may be associated with worse disease outcome rather than protective effect. However, like other studies, this study also suffers from the same limitation of low sample size. In line with the notion that antibody response is higher in severe patients, a large population study (*n* = 30,576 persons from Iceland) (Gudbjartsson et al., 2020) conducted in Iceland revealed similar observation. The study provided a comprehensive account of the relation of antibody response concerning age, sex, body-mass index, drugs habits like smoking and the use of anti-inflammatory medication. Results show that patients with smoking habit and who were on anti-inflammatory medication, had lower antibody levels, while body mass index had a positive association. The data thus suggest that antibody response may not always favor clearance of the virus, but in some instances, higher antibody levels may make the patients more vulnerable to the disease.

This detrimental relation of antibody response with poor disease outcome was also prevalent in the previous SARS-CoV infection (Zhang et al., 2006). In a study on the sera samples obtained from SARS-CoV infected patients, a faster S protein-specific antibody response was found in patients who did not survive (14.7 days), as compared with the patients who recovered from the disease (20 days). Further, the antibody titer was significantly higher in the deceased patients with faster production than in the recovered patients. To mechanistically understand why antibody response has a more detrimental effect than protective, Liu et al. (2019) studied viral antibody response in animal models (Chinese rhesus monkeys). When animals infected with the SARS-CoV and adoptively transferred with anti-S protein IgG could not prevent the infection but instead displayed severe disease symptoms. Presence of the S protein antibody abrogated wound healing, induced macrophage/monocyte infiltration into the lungs and caused the release of proinflammatory cytokine followed by acute lung injury. This study thus demonstrated that the presence of S protein-specific antibody might have a deleterious effect in inducing lung injury, irrespective of the viral load. However, since mechanistic details are difficult to discern in clinical samples, more studies in animal models need to be explored. Further, owing to the dynamics of antibody response in clinical samples concerning underlying disease condition, age, and genetic factors; animal models will provide a cleaner system to delineate the antibody dynamics with respect to disease severity (Guan et al., 2020; Hou et al., 2020).

Contrary to B cell activation, some studies have shown lower antibody durability in both mild and severe cases (Yu et al., 2020). In a longitudinal study on a 26-year-old woman with a moderate disease condition, antibody response disappeared within three months (Liu A. et al., 2020). In a sizable cohort of samples, asymptomatic patients (*n* = 37 with median age 41 years) had relatively lower durability of the IgG and IgM antibodies in comparison to the symptomatic patients (*n* = 37). Further, the viral shedding in the asymptomatic group was higher than the symptomatic group (Long et al., 2020b). Similarly, Ibarrondo et al. has shown the same antibody durability in 34 COVID-19 patients with a mean age of 43 years when studied longitudinally for a period of upto 4 months (Ibarrondo et al., 2020). The authors found a significant decline in IgG antibodies in the sera of convalescent patients with mostly mild symptoms. A declining trend was seen for multiple SARS-CoV-2 antibodies like IgG N, IgM, IgG S1, and IgA S1 in the longitudinal analysis (*n* = 487) (Gudbjartsson et al., 2020). In another longitudinal study, the disappearance of S and N protein-specific antibodies was observed within 3 months of recovery (Liu A. et al., 2020). Based on these reports, we can infer that the antibody response in some COVID-19 patients may not be long-lasting, which poses a challenge for antibody-based therapy and vaccine research—further, these data caution towards chances of reinfection, as shown to be the case with other seasonal coronaviruses (Edridge et al., 2020). However, larger cohort size and longer time frame longitudinal studies are needed to find the durability of antibody response in COVID-19.

Further, a comparison of various disease states with corresponding antibody response will provide clearer insight as to how this response is regulated. It appears that in patients with severe disease symptoms, TNF-α may influence the GC and hence B cell number (Kaneko et al., 2020), whether the same holds for asymptomatic patients with compromised antibody durability remains elusive. This dynamic antibody response is critical while considering convalescent plasma therapy (CPT) for severe or critically ill patients. If a patient already has sufficient antibodies, CPT may not be a viable treatment option (Anderson et al., 2020; Duan et al., 2020). While many studies have reported success with CPT, some studies have shown no added beneficial effects with this approach (Li L. et al., 2020). Thus, pre-caution should be taken while using this approach, i.e., if a patient already has adequate virus-specific antibodies or presence of cross-reactive and auto-antibodies, plasma therapy may do more harm than good, which may be the reason with non-responsiveness of CPT in some patients (Nagoba et al., 2020).

#### SARS-CoV-2 Antibody Cross-Reactivity and Neutralization Property

A range of SARS-CoV specific antibodies have shown cross-reactivity with SARS-CoV-2. These antibodies target S protein and mostly the RBD region (Hoffmann et al., 2020). Monoclonal antibodies against SARS-CoV such as CR3022 and S309 have shown cross-reactivity with SARS-CoV-2 (Pinto et al., 2020; Wang et al., 2020a). Similarly, in a study of 285 patients, S protein-specific antibodies from SARS-CoV showed cross-reactivity with CoV-2 N protein in a subset of patients (*n* = 5), whereas no-cross reactivity was detected against S1 subunit of SARS-CoV-2 (Long et al., 2020a). Thus, the cross-reactive nature of some of these antibodies may ensure their efficacy against multiple coronaviruses.

However, at the same time, these cross-reactive antibodies should also have neutralizing property; otherwise, they will have a harmful effect. A recent study explored the cross-reactive and neutralization property of these antibodies simultaneously (Lv et al., 2020). This study used plasma from 15 SARS-CoV-2 and 7 SARS-CoV patients and found a high degree of cross-reactivity between the antibody response from these samples, but a very low antibody neutralizing property. These results were further confirmed in animal models of SARS-CoV-2 and SARS-CoV. While S309 antibody showed better neutralization property against SARS-CoV-2, the neutralization properties for CR3022 are not yet known (Pinto et al., 2020; Wang et al., 2020a). Thus, although a high degree of cross-reactivity of the antibody response from SARS-CoV-2 can be found with other related CoVs, the neutralizing property of these antibodies may be epitope specific. The weak neutralizing property of such cross-reactive antibodies should thoroughly be tested before usage as a therapeutic intervention, to prevent the complications which may arise due to antibody-dependent enhancement (ADE). These factors also become essential while considering convalescent plasma therapy.

In an elegant recent study, Cao et al. (2020) performed sc-RNA-seq of B cells from 60 convalescent COVID-19 patients. The study led to the identification of 14 neutralizing antibodies, among which one (BD-368-2) showed the most potent effect. BD-368-2 was further explored for its efficacy in animal models and showed therapeutic potential in SARS-CoV-2 transgenic animals. Further, the study suggested the use of two different monoclonal antibodies targeting different epitopes as a more viable therapeutic intervention than a single antibody, which is impressive considering the emerging mutations in SARS-CoV-2. Thus, more research in this direction is needed to find antibodies with potent neutralization property for targeted therapy to alleviate the disease burden.

#### Antibody Dependent Enhancement in COVID-19

Non-neutralizing but cross-reactive antibodies may lead to ADE and hence enhance the immunopathological state. ADE can occur through various pathways, the most important among which include endocytosis of antibody conjugated virus by the phagocytic cells (via Fc gamma receptor IIa (FcγRIIa) and enhanced antibody immune complex formation (Kulkarni, 2020). Virus uptake by the phagocytic cells induces robust propagation and hence may further aggravate the disease condition, while antibody immune complex formation may generate a high pro-inflammatory response. Experience from previous viral infections has shown that ADE may lead to worse disease outcome in some patients with the presence of non-neutralizing antibodies, reviewed by Lee W.S. et al. (2020). In vitro studies on monocytes and macrophages have shown ADE in SARS-CoV (Flipse et al., 2016). However, no definitive clinical data is available that indicates the occurrence of ADE during SARS-CoV or SARS-CoV-2 infection. Nevertheless, based on the substantial cross-reactivity between various epitope regions of CoVs, some patients may exhibit ADE due to the presence of cross-reactive but non-neutralizing antibodies from previous infections.

#### Unconventional T Cells in COVID-19

Bronchial alveolar lavage fluid analysis of 3 COVID-19 patients reveals a high number of NKT cells during the acute phase of infection (Kim et al., 2020). This increase in NKT cells was similarly reflected in the peripheral blood. Conversely, a decline in the number of these cells was found during the recovery phase. These results thus suggest a close correlation of the NKT cell activity in COVID-19 and the presence of these cells may be required for the clearance of virus during the initial phase of infection. Concomitantly, increased infiltration and activity of these cells may lead to a more severe outcome associated with eosinophilic pneumonia, as shown in one study. However, no direct correlation of these cells types with disease severity was found, probably due to meagre sample size (*n* = 3). Further, the samples used in this study were collected at different time points after the onset of symptoms, which may have complicated the interpretation of the results.

In another study on 30 COVID-19 patients with a varied range of disease severity from mild, moderate to severe, a reduction in the total peripheral blood NKT cells was seen across groups, with no difference in the overall number between ICU (*n* = 10) and non-ICU patients (*n* = 11) (Mazzoni et al., 2020). Similarly, a study by Jouan et al. (2020) found a decrease in NKT and MAIT cells in the peripheral blood of COVID-19 patients (*n* = 30, with varied disease severity) as compared to healthy controls (*n* = 20). This decline in circulating MAIT cells was concomitantly associated with an increase in these cells in the endotracheal aspirates (ETA) obtained from critically ill patients who needed mechanical ventilation (*n* = 12), while no changes in NKT cell number in ETA were detected. The presence of circulating IL-18 reflected the activation of these cells, and the expression of PD-1 suggested subsequent exhaustion throughout the infection. This study thus indicates that the presence of the activated status of these unconventional T cells may serve as a predictive assessment of disease severity. More research about the activation, proliferation and differentiation status of these cells to the disease severity and local vs systemic effect is needed to fully understand their contribution in COVID-19 (Chen and John Wherry, 2020).

#### Lymphocytopenia During COVID-19

A drastic decrease in the number of circulating lymphocytes (lymphocytopenia) in severe and critically ill COVID-19 patients is now well appreciated (Huang C. et al., 2020; Liao et al., 2020; Liu et al., 2020a; Mathew et al., 2020; Zhou F. et al., 2020; Zhou P. et al., 2020). Interestingly, restoration in the lymphocyte count is also consistently seen during the recovery phase (Chen Y. et al., 2020). Based on these early findings, lymphocytopenia is considered a predictive indicator of COVID-19 disease severity (Tan L. et al., 2020b). Although the molecular mechanisms associated with lymphocytopenia during SARS-CoV-2 are not known, emerging evidence suggests the role of multiple factors based on the correlations drawn from previous viral infections. The decline in lymphocyte numbers in circulation can be attributed to altered chemokine and cytokine signaling responsible for the recruitment and activation/inhibition of these cells, increased infiltration to the site of infection, and cell death by apoptosis and/or necrosis (Wherry and Kurachi, 2015; Walling and Kim, 2018).

Immune profiles of COVID-19 patients show adequate levels of chemokines and cytokines involved in the maintenance of T and B cell phenotypes (Yang X. et al., 2020; Yang Y. et al., 2020). Chemokines and cytokines responsible for CD8+ T cells priming and chemotaxis were also detected in the patients. Similarly, cytokines responsible for B cell activation and proliferation signals were sufficiently present, thus excluding the possibility that lymphocytopenia may be a result of impaired activation signals or chemokine signaling. Interestingly, a recent study suggests that severely ill COVID-19 patients had lower levels of activated (CD11a+) and terminally differentiated (CD57+) peripheral blood CD4+ and CD8+ T cells (which are also S-protein reactive). The decline in the number of these cells can attribute to their concomitant migration to the infected regions under inflammatory response.

Similarly, another study has shown lymphocytopenia in peripheral blood along with a concomitant increase in the activation profile and the number of these cells in the lungs (Song et al., 2020). Homing of these activated T cells to the site of infection may thus be associated with the worsening of the disease by amplifying the proinflammatory state. A single patient analysis revealed increased CD4+ and CD8+ T cells in the BALF (Voiriot et al., 2020). ScRNA-seq in BALF followed by cluster analysis revealed the presence of CD8+ T cells with proliferative phenotype in severe cases, whereas moderate cases exhibited clonal expansion phenotype (Liao et al., 2020). From these accounts, it is indicative that increased migration of activated T cells to the site of infection may be one of the reasons for lymphocytopenia (in the blood) and the remaining T cells in the blood may eventually become dysfunctional (exhausted) as discussed below.

The decline in circulating lymphocyte number in COVID-19 patients can also attribute to the ‘exhausted’ state of these cells (Chen and John Wherry, 2020). The heightened viral load and presence of specific inhibitory signals bring about changes in the transcriptional and effector profile of T cells in a coordinated manner. Initially, they lose their property to secrete effector cytokines and gradually proceed to reduced expression of essential maintenance and activation surface receptors (Wherry and Kurachi, 2015). A subsequent increase in the expression of inhibitory receptors and associated morphological changes result in the elimination of these cells from the circulation (Wherry and Kurachi, 2015). CD4+ T cell exhaustion determines their insufficient secretion of effector molecules like IL-2, IL-10, IL-21, IFN-γ and TNF-α with a concomitant increase in inhibitory molecular signaling by PD-1, CTLA-4, LAG-3, CD244 (2B4), and TIM-3 (Blank et al., 2019; Dong et al., 2019). Similarly, CD8+ T cell exhaustion is determined by reduced expression of IL-2, IFN-γ, TNF-α, and cytolytic granules. Besides, decreased expression of T cell maintenance receptors CD122 and CD127, and increase in inhibitory receptor signaling via PD-1, CTLA-4, NKG2A, TIGIT, LAG-3, CD244 (2B4), and CD160 also mark their exhaustion (Wherry and Kurachi, 2015; Blank et al., 2019). B cell exhaustion is also demonstrated similar to T cell exhaustion with an expression of inhibitory receptors PD-1, CD22, and LAIR-1 but the exhaustion profile of these cells is relatively unexplored (Moir and Fauci, 2014).

A large body of evidence suggests functional exhaustion of CD8+ T and CD4+ T cells in the peripheral blood of COVID-19 patients. In some instances, exhaustion markers are concomitantly expressed along with activation and proliferation markers, as discussed above (Diao et al., 2020; Mathew et al., 2020; Mazzoni et al., 2020). Moreover, increased expression of exhaustion-related genes like BATF, IRF4, and CD274 also correlated with disease severity (Hadjadj et al., 2020). Interestingly, increased apoptosis of T cells became evident in severe cases as compared to mild/moderate conditions. Thus, one way to explain lymphocytopenia in COVID-19 patients is that after the onset of symptoms, T cells are primed to overcome the infection. However, in cases where viral infection persists, these cells attain robust activation, which may do more harm than good, as seen in severe and critically ill patients reviewed by Chen and John Wherry (2020). Thus, the exhaustion of these cells precedes robust activation response, and eventually, they get eliminated from the circulation, as has been seen with previous viral infections (Wherry, 2011; Blank et al., 2019). For example, during acute infection by lymphocytic choriomeningitis virus (LCMV), CD8+ T cells were shown to exhibit functional activation status and develop into memory T cells.

In contrast, during chronic infection, CD8+ T cells had impaired effector function and displayed profound exhaustion followed by apoptosis (Barber et al., 2006; Wherry et al., 2007). Similarly, CD8+ T cell exhaustion is well known during persistent human immunodeficiency virus (HIV) infection, marked by robust expression of exhaustion markers like PD-1 (Day et al., 2006; Petrovas et al., 2006). Following exhaustion, these cells are eliminated from the circulation, which is responsible for the decline in their number with long-term infection (Petrovas et al., 2009). In addition to transcriptional changes that lead to exhaustion during chronic viral infection, the presence of secretory inhibitory molecules has been implicated in lymphocyte exhaustion with a prominent role of IL-10 and TGF-β in CD8+ T cell exhaustion (Wherry, 2011; Blank et al., 2019). Increased levels of these cytokines in COVID-19 patients may also suggest their potential role in CD8+ T cell exhaustion (Chen, 2020; Liu A. et al., 2020). Furthermore, severe COVID-19 patients had elevated lactic acid levels which is a known inhibitor of T cell function (Fischer et al., 2007; Tan L. et al., 2020b).

Another vital aspect of lymphocytopenia is direct cell death by the virus during infections. HIV is a well-known example wherein CD4+ T cells undergo activation-induced cell death by the virus (Day et al., 2006; Petrovas et al., 2009). Though respiratory viruses are not known to induce T cell apoptosis directly, virus-activated secondary factors may be responsible. For example, T cell apoptosis was seen by the enhanced expression of death receptors during the infection of influenza virus (H5N1) (Boonnak et al., 2014). MERS infection was also associated with T cell apoptosis by the virus-mediated activation of intrinsic and extrinsic pathways of cell death, resulting in their depletion from circulation (Chu et al., 2014). The MERS infection was abortive in these cells, suggesting indirect activation of cell death pathways. A few in vitro studies have shown low replication of SARS-CoV in T cells and the absence of any significant cell death (Chan and Chen, 2008; Wang X. et al., 2020). Whether SARS-CoV-2 infects, T cells are currently unknown, but it appears that T cell decline during COVID-19 cannot be attributed to direct cell death by the virus but to the exhaustion mechanism.

In addition to the mechanism mentioned above associated with lymphocytopenia, secondary signaling mediated via engagement of death receptors, increased ROS, HMGB1 and other death-inducing agents released by the infected and damaged ATII cells may also be implicated in T cell decline (Kaminskyy and Zhivotovsky, 2010; Juno et al., 2017; Zhan et al., 2017). Thus, based on these early findings, lymphocyte exhaustion may be driven by multiple factors that actively engage in rendering these cells ineffective, followed by their subsequent elimination (lymphocytopenia). Overall, a clear picture is emerging, which strongly indicates lymphocytopenia as a predictive marker for COVID-19 disease severity. Along with increased neutrophil number, the blood lymphocyte count serves as a better prognostic marker and reflects the immunopathological state of the patients (Giamarellos-Bourboulis et al., 2020; Liu et al., 2020b). Further, based on these emerging studies, it is becoming evident that T cell response is heterogeneous during COVID-19 infection. While peripheral blood may exhibit lymphocytopenia, and mostly exhausted status of these cells, the site of infection is associated with an activated profile of the cells and hence determines the severity of the disease. Thus, caution should be exercised while designing therapeutic interventions for COVID-19. The underlying immunological state should be borne in mind while considering the treatment. Patients with lymphocytopenia and elevated functional and activation status of T cells may benefit from immunomodulatory approaches like mesenchymal stem cells, which are currently under clinical trials (NCT04377334). Patients with imperfect T cell and B cell responses may benefit from convalescent plasma therapy, whereas patients with impaired interferon response may respond better to interferon therapies (NCT04350671; NCT04388709). Thus, before a vaccine is available, a rational way to recommend therapy for severe cases of COVID-19 should be based on the patient’s underlying immunological state. However, the treatment options become challenging when the patients exhibit cytokine storm and associated ARDS.

Moreover, it is imperative to analyze the T and B cell response by considering the age of the patient, comorbid condition, severity score, time of sample collection, and the method used for the analysis. Because, the adaptive immune response is highly sensitive to these factors, and undermining them may thus further complicate our understanding of the development of the immunopathological state during COVID-19.

## Cytokine Storm in COVID-19 Patients

Severe and critically ill COVID-19 patients exhibit cytokine storm (CS) as a reflection of the hyperimmune activation mediated by lung resident and infiltrated inflammatory immune cells, as mentioned above. CS is manifested by the release of potent inflammatory cytokines, chemokines, and in some instances, interferons (as a late response) into the circulation that serves as an indicator of early lung damage. Though the term CS is generally used with cytokine release syndrome (CRS) during CAR-T cell immunotherapy, it can reflect different pathological conditions. While IL-6, TNF-α, and IL1-β predominantly represent CRS, CS is a much more complex response mounted by a range of inflammatory cells. Further, differences are also apparent in the kinetics, and the concentration of the cytokines released, as discussed in a recent review (Vardhana and Wolchok, 2020). Data from moderate, severe, critically ill, and recovered patients reveal a close correlation between the presence of proinflammatory cytokines with disease severity (Huang C. et al., 2020; Liao et al., 2020; Liu J. et al., 2020; Zhao et al., 2020).

Clinical evaluation of 41 COVID-19 patients (Non-ICU: 28 and ICU: 13) for over 26 chemokines and cytokines revealed increased levels of 16 of them such as IL-1β, IL-1RA, IL-17, IL-8, IL-9, IL-10, basic FGF, G-CSF, GM-CSF, IFN-γ, CXCL10, CCL2, CCL3, CCL4, PDGF, TNF-α. In comparison to non-ICU cases, patients admitted to ICU exhibited increased levels of IL1-β, IFN-γ, and IL-6, suggesting TH1 immune cell response as reported previously for SARS-CoV (Huang C. et al., 2020). Further, higher levels of G-CSF, CXCL10, CCL2, CCL3, and TNF-α indicated activation of monocytes and macrophages and damage to lung epithelial cells that were strongly correlated with ICU cases. The strength of this study is that this is the first comprehensive cytokine profiling study of the COVID-19 patients, where disease severity was compared with the cytokine response. The major limitation with this study is the small sample size for comparison between groups, and the use of lower respiratory specimen for testing rather nasopharyngeal swab sample – which is a sensitive specimen and commonly used for COVID-19 testing.

Working on relatively similar sample size (*n* = 40), Liu J. et al. (2020) found increased serum levels of IL-2, IL-6, IL-10 and IFN-γ corresponding with disease severity. By performing longitudinal analysis, IL-6, IL-10 levels were consistently increased in severe cases (*n* = 13). Besides small sample size, the major limitation with this study was that most of the patients had a comorbid condition like diabetes, hypertension, fungal infection and other chronic ailments, which may complicate the data interpretation and comparison between groups. By working on a relatively larger cohort (*n* = 799), Chen T. et al. (2020) found that increased levels of IL-2R, IL-6, IL-8, IL-10, TNF-α in patients who had to succumb to the disease (*n* = 113) as compared to those who recovered (*n* = 161). However, unlike the study by Huang et al. IL-1β was not found to be significantly increased in deceased patients. This discrepancy could be because the samples used by Huang C. et al. (2020) were from critically ill (5 deceased out of 13) patients, whereas Chen et al. analyzed deceased patients. Thus, observed differences in levels of cytokines may reflect the disease severity and underlying comorbid condition.

In addition to the peripheral blood profiles, transcription profiling of BALF revealed higher expression of IL-10, CCL2, CXCL10, CCL3, and CCL4, a representation of lung immunopathology. (Xiong et al., 2020). Similarly, in another study measurement of IL-1β, IL-2, IL-4, IL-5, IL-6, IL-8, IL-10, IL-12p70, IL-17, IFN-α, IFN-γ, and TNF-α were performed in the BALF, and the results revealed a significant increase in IL-1β, IL-6, and IL-8 levels in critically ill patients as compared to moderate condition (Liao et al., 2020). BALF fluid analysis of 8 COVID-19 patients by Zhou Z. et al. (2020) also showed upregulation of key chemokine transcripts that are involved in the recruitment of inflammatory cells (IL1RN, IL1β, CXCL17, CXCL8, CXCL1, CXCL2, CCL2, and CCL7) (Zhou Z. et al., 2020).

ScRNA-seq analysis of BALF also revealed increased expression of CXCL9, CXCL10, CXCL11, and CXCL16 in all tested COVID-19 patients (Liao et al., 2020). Lung macrophages displayed increased transcripts of IL-1β, IL-6, TNF-α along with chemokines like CCL2, CCL3, CCL4, and CCL7 in severe cases. In terms of the contribution by the lung resident cells, tissue immunohistochemistry data revealed increased levels of IL-6, TNF-a and IL-10 in the AMs of biopsy samples obtained from deceased patients (Wang et al., 2020b). Together, these studies unequivocally show heightened proinflammatory cytokine and chemokine response in circulation as well as at the site of infection in severe and critical cases. Since these initial reports, all the subsequent studies revealed a consistent increase in IL-6, and to some extent TNF-α (Chen G. et al., 2020; Diao et al., 2020; Luo et al., 2020; Qin et al., 2020; Tan M. et al., 2020c; Zheng M. et al., 2020). Based on the clinical cytokine profile across the studies in critical/deceased patients, serum IL-6 level is well established as a reliable predictive marker for COVID-19 severity and a potential marker of ARDS along with increased neutrophil/lymphocyte ratio. Thus, considering a robust increase in IL-6 levels in the majority of the severe COVID-19 patients, currently, treatments are underway to lower the levels of this pleiotropic cytokine. For example, antibodies directed against IL-6R (such as tocilizumab) have shown promising clinical outcomes (Capra et al., 2020; Luo et al., 2020; Xu X. et al., 2020). Similarly, antibodies directed against GM-CSF (NCT04351243) and IL-β (NCT04348448) are also being explored for their efficacy to attenuate CS in COVID-19 patients. A detailed list of the recent papers which reported clinical profiles of these inflammatory molecules in COVID-19 patients is provided in Table 1.

**TABLE 1:** List of some research articles from December 2019 to May 2020 establishing lymphocytopenia and cytokine storm in COVID-19 patients.

**S. No**	**No. of patients**	**Findings**	**Sample**	**References**
1.	Total: 40 Mild: 27 Severe: 13	• Decrease in CD8^+^ and CD4^+^ T cell counts were observed suggesting lymphocytopenia • Increase in IL-2, IL-6, IL-10, and IFN-γ levels in severe cases • No significant changes were observed in IL-4 and TNF-α levels • The number of T cells increased in patients who recovered from the disease, along with a decrease in the cytokine levels comparable to mild cases	Peripheral blood/Serum	(Liu J. et al., 2020)
2.	Total: 147 Healthy control: 45 Mild/moderate: 42 Severe: 43 Critical: 17	• Increased levels of serum cytokines like TNF-α, IFN-γ, IL-2, IL-4, IL-6 and IL-10 were found in all COVID-19 patients • Similarly, CRP levels were increased in all COVID-19 patients, which showed positive correlation with IL-10 • **IL-6** and **IL-10** levels were suggested as predictive disease severity biomarkers	Serum	(Han et al., 2020)
3.	Total:138 Non-ICU: 102 ICU: 32	• Ninety-seven patients from both the groups showed lymphocytopenia. • Neutrophil count was significantly higher in ICU patients. • ICU patients showed significantly elevated levels of D-dimer, creatine kinase–MB, LD, ALT, AST, and procalcitonin suggesting multiple organ dysfunction in ICU cases.	Peripheral blood/Serum	(Wang D. et al., 2020)
4.	Total:191 Recovered: 137 Deceased: 58	• Deceased patients had lower levels of lymphocyte and platelet count. Whereas higher levels of ALT, LDH, creatinine, creatinine kinase, troponin I, Serum ferritin, and D-dimer was observed in deceased patients’ samples • **d**-DIMER was suggested as a potential marker for COVID-19 severity • Higher IL-6 levels were found in deceased patients	Peripheral blood/Serum	(Zhou F. et al., 2020)
5.	Total:41 Non-ICU: 28 ICU: 13	• Decrease in total lymphocyte count while increase in neutrophil count was observed in ICU patients. • Higher plasma levels of IL-2, IL-7, IL-10, GCSF, IP-10 (CXCL10), CCL2, CCL3, and TNF-α were observed in ICU patients as compared to non-ICU. • Increased levels of T_*H*_2 cytokine IL-4 was reported. • Increased levels of D-dimer, ALT, and AST in ICU patients.	Peripheral blood/Serum	(Huang C. et al., 2020)
6.	Total:150 Recovered: 82 Deceased: 68	• Increased levels of absolute lymphocyte count. • Significantly increased levels of IL-6, blood creatinine, myoglobin, cardiac troponin, CRP, total bilirubin, and blood urea nitrogen were observed in the deceased patients. Further, higher levels of ALT, AST, LDH, creatinine, and creatinine kinase were observed.	Peripheral blood/Serum	(Ruan et al., 2020)
7.	Total:50 moderate: 14 Severe: 25 Critically ill: 11	• Total percentage CD4^+^ T and CD8^+^ T cells was significantly lower in the severe cases along with increase in total neutrophil percentage, indicating overall dysfunctional immune response. • Increased levels of IFN-γ, IL-1ra, IL-2ra, IL-6, IL-10, IL-18, HGF, CCL7, MIG, M-CSF, G-CSF, MIG-1a, CTACK, and IP-10. • IP-10, CCL7, and IL-1RA were higher in severe cases compared to moderate. • Lymphocytopenia and increased neutrophil count was suggested to correlate with disease severe.	Peripheral blood/Serum	(Yang Y. et al., 2020)
8.	Total:21 moderate: 10 Severe:11	• Absolute count of lymphocytes was decreased and specifically levels of CD4^+^ T and CD8^+^ T were lower in severe cases. • Higher levels of IL-2R, IL-6, IL-10, and TNF-α were observed in severe cases. • Higher levels of ALT, LDH, CRP, ferritin, and D-dimer was detected in severe cases. • Further, levels of IFN-γ levels specifically measured in CD4^+^ T cells were lower in severe cases.	Peripheral blood/Serum	(Chen G. et al., 2020)
9.	Total:1 deceased	• CD4^+^ T and CD8^+^ T cells were reduced, however, they exhibited activated status. Higher cytotoxic granules in CD8^+^ T cells indicated overactivation. • Increased levels of T_*H*_17 cells. • Inflammatory mononuclear infiltrates were observed in the lung tissue.	Peripheral blood/Serum	(Xu Z. et al., 2020)
10.	Total: 452 Non-Severe: 166 Severe: 286	• The total number of T cells were decreased in severe case. Further lower levels of CD4^+^ regulatory and CD8+ suppressor T cells was observed in severe cases. • Severe cases exhibited higher neutrophil counts and an increase in NLR ratio. Whereas blood monocytes and eosinophil counts were lower. • Among the proinflammatory markers, increased levels of TNF-α, IL-2R, and IL-6, IL-8, IL-10 along with CRP, serum ferritin, and procalcitonin was observed. Whereas no significant difference in IL-1β was found.	Peripheral blood/Serum	(Qin et al., 2020)
11.	Total: 56 Mild/Moderate: 31 Sever: 25	• The levels of CD4^+^, CD8^+^ T cells, NK cells, and B cells were lower in severe cases. Whereas, T_*REG*_ cells were found to be moderately increased in mild cases. • Higher levels of IL-2, IL-6, IL-10, and TNF-α were found in severe cases. Increase in IL-4 was observed in mild but not severe cases. Further, **IL-2** and **IL-6** were suggested as reliable indicators for disease severity. • Serum leves were inconsistent with low levels or unchanged in COVID-19 patients.	Peripheral blood/Serum	(Tan M. et al., 2020c)
12.	Total: 222 Non-severe: 81 Severe: 67	• Higher NLR with lower levels of CD4^+^, CD8^+^ T cells in severe cases. • Higher cytokine levels of IFN-γ, IL-2, IL-6, and IL-10 was found in patients with high NLR. No significant difference in IL-4 levels were observed between severe and non-severe cases. • **NLR** was suggested as a predictive disease marker	Peripheral blood/Serum	(Zhang et al., 2020a)
13.	Total: 25 Recovered: 14 Non-recovered: 11	• Lower levels of CD3^+^ T cells, CD4^+^ T cells, CD8^+^ T, NK cells as well as B cells were found in COVID-19 patients. Treated patients who exhibited clearance of virus showed restoration in levels of CD3^+^ T cells, CD4^+^ T cells, CD8^+^ T cells, whereas NK cell count was inconsistent. • **Lymphocytopenia** was considered as predicative biomarker for the disease.	Peripheral blood	(Chen X. et al., 2020)
14.	Total: 22 Healthy: 10 Mild/Moderate: 10 Severe: 6	• Marked reduction in functional CD4^+^ T cells was observed, as revealed by decrease in IFN-γ, and TNF-α produced by these cells in severe group than mild. CD8+ T cells revealed activation profile as marked by increased levels of granzyme B and perforin in severe than mild cases. • CD8+ cells had increased expression of exhaustion marker CTLA-4 in severe than mild and increased PD-1 in mild than healthy group. • No significant differences in IL-6 and TNF-α was observed between severe, mild and healthy controls. However, an increasing trend was observed.	Peripheral blood/Serum	(Zheng H.Y. et al., 2020)
15.	Total: 68 Mild/Moderate: 55 Severe: 13	• Decreased number of CD8^+^ T and NK cells in patients showing severe symptoms. • Decrease in expression of IFN-γ, IL-2, granzyme-B by CD8^+^ T, and decrease in TNF-α expression by NK cells was observed, indicating CD8^+^ T and NK cell exhaustion.	Peripheral blood/Serum	(Zheng M. et al., 2020)
16.	Total: 99 patients with mild to severe symptoms	• Decrease in lymphocyte count and increase in neutrophils was observed when compared to the normal range. • Increased serum levels of IL-6, D-dimer, ALT, AST, LDH, Myoglobin, Procalcitonin, Serum ferritin, Erythrocyte sedimentation rate, and CRP was observed in all the studied patients. Serum creatinine, and creatinine kinase showed inconsistent trend.	Peripheral blood/Serum	(Chen N. et al., 2020)
17.	Total: 522 Mild/moderate: 151 Severe: 53	• Decreased number of total CD4^+^ and CD8^+^ T cells were observed in most of the patients with further decrease reported in patients who were admitted to ICU. • T cell exhaustion markers like PD1 and TIM3 were higher in severe disease patients. • Serum levels of TNF-α, IL-6 and IL-10 were higher in symptomatic patients. However, no significant changes in the levels of IFN-γ, IL-2, and IL-4 were observed across groups. • T cell count restored and levels of IFN-γ, IL-10, IL-6, and TNF-α decreased as the disease resolved in a subset of patients.	Peripheral blood/Serum	(Diao et al., 2020)
18.	Total: 710 Survivor: 20 Non-survivors: 32	• Blood analysis of non-survivors revealed decreased total lymphocyte count, and increase in platelet count. Serum analysis revealed increase in total bilirubin, creatinine, and lactate concentration.	Peripheral blood/Serum	(Yang X. et al., 2020)
19.	Total: 34 patients with varying disease severity	• Presence of increased levels of inflammatory monocytes in the blood which were found to secrete proinflammatory cytokines like IL-10, IL6, and TNF-α.	Peripheral blood/Serum	(Zhang D. et al., 2020)
20.	Total:80 Non-severe: 11 Severe: 69	• The levels of CRP, ferritin, IL-6 and LDH were shown to be associated with longer treatment modality. • **IL-6** levels were shown to positively correlate with disease severity and suggested as a predictive biomarker.	Serum	(Liu T. et al., 2020)
21.	Total:36 Healthy: 10 Non-ICU: 16 ICU: 10	• Significant decrease in total lymphocytes including CD4^+^ and CD8^+^ T cells was observed in ICU patients as compared to non-ICU. Both CD4^+^ and CD8^+^ T cells exhibited activated phenotype. However, no changes were observed in B cells and NK cells between ICU and non-ICU patients. • Higher percentage of inflammatory monocytes were detected in COVID-19 patients, with further increase in ICU patients. These monocytes were shown to secrete higher levels of GM-CSF and IL-6.	Peripheral blood	(Zhou et al., 2020a)
22.	Total:799 Recovered: 161 Deceased: 113	• Leukocytosis was observed in 56 patients who died due to the disease. Deceased patients had robust decrease in lymphocytes and displayed lymphocytopenia. • Levels of IL-2, IL-6, IL-8, IL-10, and TNF-α were higher in deceased as compared to the recovered patients. • Serum levels of D-dimer, ferritin, ALT, AST, procalcitonin, creatinine creatine kinase, total bilirubin, ALP, and GGT were markedly increased in deceased patients.	Peripheral blood/Serum	(Chen T. et al., 2020)
23.	Total: 70 Moderate- cured: 40 Severe-cured: 15 Death: 15	• Robust decline in lymphocyte percentage in blood of patients who died due to the disease. Whereas, substantial recovery of the blood lymphocyte percentage was seen in the patients who recovered from the disease. • The study suggests **lymphocytopenia** as a predictive prognosis marker for COVID-19.	Peripheral blood	(Tan L. et al., 2020b)
24.	Total: 70 Mild/Moderate: 8 Critical: 10 Control: 10	• Serum levels of CCL5 was highly increased in critically ill patients as compared to mild/moderate and healthy control. • Levels of IL-1β, IL-6, IL-8, and other chemokines CXCL8, CCL4, CCL3 were also increased.	Serum	(Patterson et al., 2020)
25.	Total: 54 Mild/Moderate: 26 Severe: 28	• Macrophage activation syndrome along with hyperactivation of monocytes was found in severe cases. • Decrease in lymphocyte number (CD4^+^T and B cells) along with decline in NK cells was observed. • Blood IL-6 and CRP levels were increased in severe cases. Further, increased levels of fibrinogen, D-dimers, creatinine was found in severe cases.	Peripheral blood/Serum	(Giamarellos-Bourboulis et al., 2020)
26.	Total: 48 SARS-CoV-2 positive: 24 SARS-CoV-2 negative: 24	• Reduced IFN-I and IFN-III response was observed in patient samples as well as in *in-vitro* cell culture model of primary respiratory epithelial cells. • Increase in serum levels of IL-6, IL1RA, CCL2, CCL8 CXCL2, CXCL8, CXCL9, and CXCL16 were observed in	Serum	(Blanco-Melo et al., 2020)
27.	COVID-19: 1484 Healthy: 9 Others: 272	• Significantly higher levels of IL-6, IL-8, and TNF-α were observed in serum of COVID-19 patients as compared to the healthy control or patients with multiple myeloma. • **IL-6, IL-8,** and **TNF-α** were associated with worst disease outcome and suggested as the predictive indicators of the disease.	Serum	(Del Valle et al., 2020)
28.	Total: 102 COVID-19: 27 Flu: 75	• COVID-19 patients showed increased levels of serum CRP, which was associated with disease progression. • **CRP** levels were suggested as a predictive marker in early stage COVID-19.	Serum	(Tan C. et al., 2020)
29.	Total: 132 Mild/moderate: 60 Severe: 56 Critical: 16	• Decreased lymphocyte number was observed in severe and critically ill patients. Similarly, increased levels of serum CRP and SAA was observed. • **SAA** along with **lymphocytopenia** was suggested as a predicative disease marker.	Peripheral blood/Serum	(Li H. et al., 2020)
30.	Total: 377 Mild/moderate: 260 Severe: 117	• Increased NLR, along with increased levels of serum CRP and D-dimer were observed in patients with severe disease symptoms. • **NLT, CRP** and **d**-DIMER were suggested as predictive disease markers.	Peripheral blood/Serum	(Zhou et al., 2020b)
31.	Total: 377 Mild/moderate: 69 Severe: 24	• Significantly higher NLR, PLR, LMR, and total neutrophil count was observed in severe cases. Whereas, the count of lymphocytes was decreased. • Serum CRP levels were higher in severe cases. • **NLR** was suggested as predictive disease biomarker.	Peripheral blood	(Yang A.P. et al., 2020)
32.	Total: 18 Severe: 16 Critical: 2	• Increased activation profile of CD4^+^, CD8^+^ and plasmablasts was seen in COVID-19 patients. • Interestingly severe cases were associated with activated profile of CD4+ T cells, lower number of T_*FH*_ cells and exhaustive CD8+ T cells. • Increased levels of serum AST, CRP, and creatinine kinase was observed. • **CRP** was suggested as the predictive disease marker.	Peripheral blood/Serum	(Wang G. et al., 2020)
33.	Total: 221 Normal: 36 Recovered: 60 COVID-19: 125	• CD4+ and CD8+ T cells were associated with the expression of activation markers (CD38 and HLA-DR), proliferation markers (Ki-67), and exhaustion markers (PD-1). • This elaborate study characterized 200 immune parameters and correlated with clinical features and disease severity. • Increase in chemokines like CXCL10, CXCL9, CCL2, and IL-1RA were observed in half of the COVID-19 patients. • Increased levels of ferritin, D-dimer, and CRP were observed in COVID-19 patients as compared to healthy controls or recovered patients.		(Mathew et al., 2020)
34.	Total: 41 Mild: 29 Severe: 12	• Slight lymphocytopenia was observed in mild patients and significantly higher in severe COVID-19 patients as revealed by decrease in CD4+, CD8+ and NK cell number. • Peripheral blood showed increased activation status of CD8+ T with higher percentage of CD38^+^CD8^+^, CD38^+^HLA-DR, and CD38^+^HLA-DR^+^CD8^+^ cells in severe than mild cases. While no significant changes were seen in CD4^+^ • Increased expression of exhaustion markers PD-1 and TIM-3 was observed in CD8^+^, and PD-1 in CD4^+^ T cells in case of severe cases than mild. • Increased levels of IL-6, TNF-α, IL-17A, IFN-γ, MCP-1, IL-10, IL-4, and IL-5 were observed in COVID-19 patients.	Peripheral blood	(Song et al., 2020)
35.	Total: 82 (deceased)	• Lymphopenia, neutrophilia, and thrombocytopenia was observed. • Increased levels of CRP, LDH, ALT, and D-dimer were found. • Increased levels of IL-6	Peripheral blood	(Zhang et al., 2020b)
36.	Total:24 Mild: 4 Severe: 5 Critical: 3	• Innate immune cells like monocytes, NK cells and myeloid-derived suppressor cells increased from mild to severe cases than declined in the critically ill patients. • CD8+ T cell subsets were decreased in all the disease groups. However, CD4+ T, naïve CD4+ T, and TGF-β+CD28- naïve CD4+ T cells were increased. On the contrary memory CD4+ T cells were decreased. • Levels of functional molecules such as CXCR3, CD28, and TGF-β were increased in patients as compared to health control.	PBMCs	(Wang W. et al., 2020)
37.	Total: 53 Moderate: 21 Severe: 18 Critical: 14	• CD11a expressing lymphocytes (CD4^+^ and CD8^+^) and B cells were decreased in critically ill patients. Interestingly, it was suggested that T cell decline in circulation may be associated with increased infiltration of these cells toward the site of infection. Whereas recovered patients showed reduced decline in CD11a T cells. Thus, **CD11a** positive T cells were suggested as a possible prognostic marker of COVID-19. • Interestingly, eosinophil number was increased patients who were critically ill.	PBMCs	(Anft et al., 2020)
38.	BALF Healthy: 3 Patients: 2 PBMCs Healthy: 3 Patients: 3	• RNA-seq analysis was performed. • Increased transcript levels of IL-10, CCL2/MCP-1, CXCL10/IP-10, CCL3/MIP-1A, and CCL4/MIP1B in BALF. • Increased transcript levels of CXCL10, TNFSF10, TIMP1, C5, IL18, AREG, NRG1, IL10 in PBMCs.	PBMCs and BALF	(Xiong et al., 2020)
39.	Total: 12 Healthy: 3 Moderate: 3 Severe/critical: 6	• Single cell RNA-seq analysis was performed followed by cluster analysis to determine the immune cell type. • Proliferative and less expandable CD8^+^ T lymphocytes were present in severe/critically ill patient BALF samples, whereas moderate cases showed more expandable and less proliferative phenotype. • Increased macrophages and neutrophils were detected in the BALF of patients exhibiting severe/critical disease symptoms. • Higher levels of IL-1β, IL-6, and IL-8 were observed in all the patients. • Increased levels of CXCL9, CXCL10 and CXCL11 were consistently seen in all the patients. • Lung macrophages displayed higher transcripts of IL1B, IL6, TNFA, along with chemokines CCL2, CCL3, CCL4 and CCL7 in severe cases.	BALF	(Liao et al., 2020)
40.	Total: 174 Healthy: 20 COVID-19: 8 Others: 146	• Transcript levels of IL1RN, IL1B, CXCL17, CXCL8, CXCL1, CXCL2, and CCL2, CCL7 were increased in COVID-19 patients in comparison to healthy controls. • Upregulation of calgranulin genes S100A8, S100A9 and S100A12 were observed. • Interferon response was also observed as indicated by upregulation of about 83 ISGs in COVID-19 patients.	BALF	(Zhou Z. et al., 2020)

*The highlighted studies show the respective predictive biomarkers (in bold) which were found in the study.*

## Acute Respiratory Distress Syndrome

The consequences of the dysfunctional immune response as characterized by increased inflammatory cell infiltration, cytokine storm, and lymphocytopenia are the underlying concerns in COVID-19 patients. This immunopathological state may eventually lead to the development of ARDS, which is associated with between 67 and 85% of ICU deaths (Ji et al., 2020; Liu et al., 2020b). With a median time range from 8 to 12 days after symptom onset, ARDS progresses hierarchically during COVID-19 (Fan E. et al., 2020).

**FIGURE 5:**
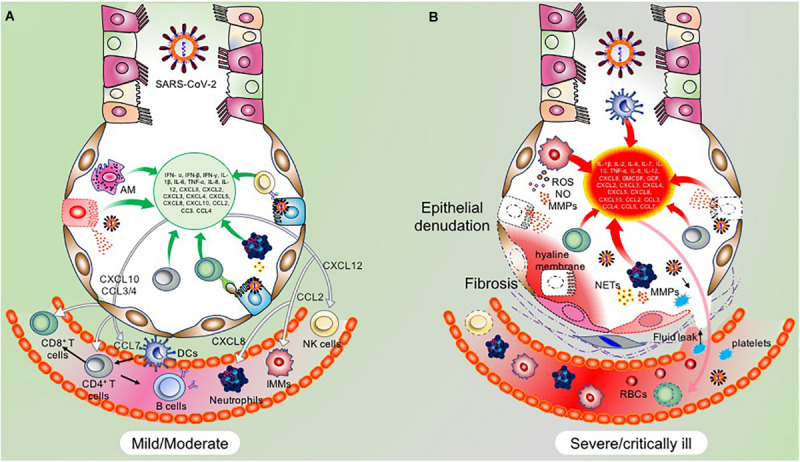
Immunopathological changes during different stages of COVID-19. **(A)** Immunological response in mild/moderate COVID-19 patients are overtly conferred by the adaptive immune cells with assistance from the innate immune system. Infected ATII cells and activated AMs produce a repertoire of cytokines and chemokines to recruit innate and adaptive immune cells and limit the viral propagation. The functional immune system thus acts in a well-coordinated manner to eliminate the virus specific ATII cells. Due to the relatively stem cell-like property of ATII cells, the eliminated cells are subsequently regenerated, thus ensuring recovery of the damaged lung tissue. **(B)** However, in severe/critically ill patients, an exaggerated inflammatory response is mounted by hyperactivated innate immune cells, and to a lower degree by adaptive immune cells. A hyperinflammatory state is created in the lungs which is characterized by the robust accumulation of inflammatory cells like monocytes/macrophages, dendritic cells, and neutrophils. This leads to the excessive release of cytokines and chemokines by these cells, thus inducing a vicious hyperinflammatory cycle. Damage to the lung parenchyma is inflicted by this hyperinflammatory state, along with other cytotoxic molecules like MMPs, NETs, ROS and NO. The latter two combine to form more cytotoxic peroxynitrite ions. The combined action of these events results in epithelial denudation, vascular leak, platelet and RBC infiltration, vascular edema, and hyaline membrane formation, resulting in ARDS.

The late robust immune response generated by excessive infiltration of innate immune cells, proinflammatory cytokines, along with activated lymphocytes causes extensive damage to the lungs. By this time, the injury incurred by the hyperactivated innate immune system far bypasses the damage by the virus, eventually leading to respiratory collapse and multi-organ failure. Taking cues from animal models and post-mortem samples of deceased SARS-CoV infected patients, a comprehensive picture of the clinical and molecular pattern of lung damage has emerged (Leung et al., 2005) as shown in Figure 5. These events proceed in a sequential and well-coordinated manner. Excessive alveolar epithelial cell death, loss of extracellular matrix, deposition of cellular debris, and formation of hyaline membrane starts early along with bronchiolar and alveolar epithelial cell denudation, referred to as diffuse alveolar damage (DAD). These events are proceeded by desquamation of the alveolar and bronchial epithelial lining, altered permeability of the alveolar-vascular barrier, interstitial pulmonary edema, thrombosis, coagulation, and fibrin deposition (Gralinski et al., 2013). Finally, the SARS-CoV disease progresses to a more severe fibrotic stage with increased fibroblast cell proliferation, interstitial fibrosis, collagen deposition, and complete collapse of the airways. The contents are released to the secondary tissues along with the proinflammatory cytokines, which may result in multi-organ failure. These changes are orchestrated by a coordinated action of molecular events that are ever-expanding (Chong et al., 2004; Jih, 2005; Yen et al., 2006).

Recovery from ARDS is possible but only before the disease progresses to a critical stage. However, a mortality rate between 35 and 40% in ARDS patients demonstrates irreversible tissue damage manifested by tissue fibrosis (Bellani et al., 2016). TGF-β plays a central role in the late onset of fibrotic changes during ARDS. Acting with other proinflammatory molecules, TGF-β induces fibroblast cell proliferation and activation, collagen synthesis and deposition, synthesis of fibronectin, and alpha-smooth muscle actin, which all together contribute to fibrosis (Fan E. et al., 2020). Emerging histological studies from deceased COVID-19 patients reveal robust structural changes in the lungs and associated organs like the liver, kidneys, and heart (George et al., 2020). Lung biopsy samples from a 50-year-old patient who had succumbed to COVID-19 revealed hyaline membrane formation, desquamation of epithelial and endothelial cells, mononuclear infiltration, and robust ATII cell damage, indicating ARDS (Xu Z. et al., 2020).

Further, microvesicular steatosis was observed in liver biopsy samples suggesting multi-organ damage (Xu Z. et al., 2020).

Similarly, histopathological examination of a 72-year old patient who died due to SARS-CoV-2 infection revealed inflammatory infiltrates and fibrosis in the lungs along with intra-alveolar organizing fibrin suggesting ARDS (Zhang H. et al., 2020). In another study, lung samples from 38 deceased COVID-19 patients were evaluated for histopathological changes. Necrosis of alveolar epithelial cells and DAD was observed in all patients. While, most of the patients exhibited interstitial and intra-alveolar edema, ATII cell hyperplasia, thrombosis, fibrin deposition, infiltration of macrophages and lymphocytes, hyaline membrane formation and other fibrotic changes (Carsana et al., 2020; Polak et al., 2020). Similar histopathological findings were revealed by other studies (Menter et al., 2020).

Clinical evaluation of 113 deceased patients revealed ARDS and multi-organ dysfunction (Chen T. et al., 2020). Increased serum concentration of molecular markers such as D-dimer, cardiac troponin creatinine, creatine kinase, alanine aminotransferase, aspartate aminotransferase, total bilirubin, alkaline phosphatase, and γ-glutamyl transpeptidase was observed. Presence of these molecules indicates damage to the liver, kidneys, and heart. Further, thrombosis and fibrinolysis were seen across the studies as revealed by elevated serum D-dimers. Besides, clinical evaluation of critically ill and deceased patients demonstrated elevated levels of biomolecules associated with damage to lungs, liver, kidneys, and heart, pointing to multi-organ dysfunction during COVID-19 (Chen T. et al., 2020; Huang C. et al., 2020; Zhu et al., 2020). It is emerging that ARDS in COVID-19 patients have a consistent presence of hyaline membrane, DAD, thrombosis and fibrotic changes, which may be the primary cause of ARDS-induced death (Fan E. et al., 2020; Price et al., 2020). Prospective longitudinal studies on clinical, molecular, and associated histopathological parameters will further delineate the molecular basis of ARDS in COVID-19. A list of studies that have detected D-dimers and associated clotting factors in COVID-19 patients is presented in Table 1.

## Future Perspectives

Over the last several months, a large number of clinical and histological studies have illustrated the underlying pathophysiological changes and tissue damage in COVID-19. However, we are just beginning to understand the fundamental molecular and signaling pathways implicated in this disease pathogenesis. Close sequence similarity with SARS-CoV does help us understand some co-existing pathological features but owing to reasonable genomic and structural variations, and it is essential to decoding the molecular mechanisms specific to SARS-CoV-2 infection. Differences in S protein, ORF3b, ORF6 and ORF8 between SARS-CoV and SARS-CoV-2 are functionally relevant (Chan et al., 2020; Mantlo et al., 2020). Similarly, the differential immune responses generated by the two viruses needs to be delineated well to develop targeted therapies to modulate these specific molecular networks (Moreno-Eutimio et al., 2020; Yao et al., 2020). Importantly, heterogeneous T cell response in COVID-19 patients has remained an enigma, with lymphocytopenia and activated T cell state in some patients versus an increased presence of exhausted T cells in others.

Further, the increased activation status of these cells at the site of infection (lungs) in severe cases adds more complexity to the T cell immune response in COVID-19 patients and hence may pose difficulty in devising a universal therapeutic intervention. Similarly, the presence of reactive T cells in healthy individuals is another area that needs a comprehensive understanding, mainly while designing a vaccine. Keeping these challenges in mind and till the time an effective vaccine becomes available, existing immunomodulatory approaches like mesenchymal stem cell-based therapies (currently under clinical trials), anti-IL6 and anti-GMCSF drugs to counter cytokine storm, as well as antiviral drugs remain the standard therapeutic interventions. While the antiviral drug remdesivir has shown promise in some patients, severe side effects were also reported in others (Wang Y. et al., 2020). Considering the number of factors that affect the complexity of immune response during COVID-19, it is crucial to understand that a single type of intervention may not work for all patients. Thus, it appears that exploring a combination therapy may be more compelling at present. However, determination of the optimal combination, dose, and time of treatment needs thorough investigation. These targeted therapies become critical, considering the chance of reinfection. A recent study on ten healthy individuals throughout 35-years revealed short-lasting immunity against four common seasonal coronaviruses, with chances of reinfection in a year after infection (Edridge et al., 2020). It is plausible that SARS-CoV-2 may exhibit the same tendency of reinfection, which may be a growing concern for vaccine research. Thus, a more comprehensive understanding of the immunopathological changes and sustainability of protective immunity is needed. In this review, we highlight some of these immunological responses, which are central to the progression and outcome of COVID-19 patients. Ongoing research in this direction should lead to effective therapies sooner rather than later, alongside with the vaccines.

## Author Contributions

TA conceptualized the idea, generated the figures, and wrote the manuscript. RC helped in writing the immunology section. SA helped in designing and writing the ARDS section. MJ helped in writing the microbial nucleic acid-sensing and signaling part. AA ad AR helped in writing the innate immune response in SARS-CoV-2. All authors contributed to the article and approved the submitted version.

## Conflict of Interest

The authors declare that the research was conducted in the absence of any commercial or financial relationships that could be construed as a potential conflict of interest.

## Abbreviations

ALP: alkaline phosphatase
ALT: alanine aminotransferase
AREG: amphiregulin
AST: aspartate aminotransferase
BALF: bronchoalveolar Lavage Fluid
CCL: Chemokine (C-C motif) ligand
CD: cluster of differentiation
CRP: C-reactive protein
CTACK: (cutaneous T-cell -attracting chemokine)
CXCL: chemokine (C-X-C motif) ligand
G-CSF: Granulocyte colony-stimulating factor
GGT: γ -glutamyl transpeptidase
GM-CSF: Granulocyte-macrophage colony-stimulating factor
IFN: Interferons
IL: Interleukins
ISG: Interferon stimulating gene
LD: lactate dehydrogenase
LDH: Lactate dehydrogenase
LMR: lymphocyte-to-monocyte ratio
MCP: Monocyte Chemoattractant Protein
MIP: Macrophage Inflammatory Protein
NK: Natural killer
NLR: neutrophil/lymphocyte ratio
NRG: Neuregulin
PBMC: peripheral blood mononuclear cell
PD1: Programmed cell death protein 1
PLR: platelet-to-lymphocyte ratio
SP: Surfactant protein
SSA: Serum Amyloid A
TIM3: T-cell immunoglobulin and mucin-domain containing-3
TIMP1: Tissue inhibitor of metalloproteinases
TNF: Tumor necrosis factor
TNFSF10: (tumor necrosis factor (ligand) superfamily, member 10).
