# Supplementary material for: COVID-19: The Emerging Immunopathological Determinants for Recovery or Death
Source: Front Microbiol. 2020 Dec 1;11:588409. doi: 10.3389/fmicb.2020.588409 (PMC7736111; doi:10.3389/fmicb.2020.588409)
Supplement: Supplementary file 1 [file Table_1.docx]

**Supplemental Table 1: Reported host cell targets of SARS-CoV and presumably of SARS-CoV-2 to evade host cell sensing and inhibit early immune response**

| **Virulent factor** | **Effect on host cells** | **Target** | **Virus** | **REF** |
| --- | --- | --- | --- | --- |
| Nsp1 | Inhibits 40s ribosomal subunit loading and induces degradation of host cell mRNAs. | 40s subunit | SARS CoV | (Huang et al., 2011; Tanaka et al., 2012) |
|  | Inhibits STAT1 phosphorylation and transcription of ISGs and also strongly inhibits IFN-β promoter activity. | STAT1 | SARS CoV | (Wathelet et al., 2007) |
|  | Suppressed IFN-β, ISG-15 and ISG56 synthesis | unexplored | SARS CoV | (Narayanan et al., 2008) |
| Nsp2 | Possibly inhibiting translational initiation of host cells and | ELF4E2, FKBP15 | SARS-CoV-2 | (Gordon et al., 2020) |
| Nsp3  also, PLpro | Inhibits IFN signaling by interacting with TRAF3, TBK1, IKKε, STING, complex. | TRAF3, TBK1, IKKε, STING, and IRF3 | SARS-CoV | (Chen et al., 2014) |
|  | Activates E3 ligase RCHY1 which stimulates ubiquitination of p53 followed by its degradation. | E3 ligase RCHY1 |  | (Ma-Lauer et al., 2016) |
|  | PLP2 induces degradation of p53 and further inhibits its transcriptional levels. | MDM2 |  | (Yuan et al., 2015) |
|  | PLpro inhibits IFN signaling by directly interacting with IRF3 and preventing its phosphorylation | IRF3 |  | (Devaraj et al., 2007) |
|  | Blocks phosphorylation of IRF3 and hence inhibits its nuclear translocation and secretion of IFN-β. Similarly, blocks NF‑κB signaling. | Unexplored |  | (Frieman et al., 2009) |
| Nsp4 | Nsp4 may be implicated in the regulation of nuclear pore complex, but the exact role is not known. | NUP210 | SARS-CoV-2 | (Gordon et al., 2020) |
| Nsp5  also  3CL^pro^ | Induces apoptosis in a caspase 3 and caspase 9 dependent manner in human promonocyte cell line. | Unexplored | SARS-CoV | (Lin et al., 2006) |
|  | NSP5 may have a probable role in interfering with IFN response and inflammatory pathway. | HDAC2 | SARS-CoV-2 | (Gordon et al., 2020) |
| Nsp6 | Probable role in regulating ion transport in host cells. | ATP6AP1, Sigma-1 receptor | SARS-CoV-2 | (Gordon et al., 2020) |
| Nsp7 | Inhibits IFN-β signaling | Unexplored | SARS CoV | (Frieman et al., 2009) |
|  | Multiple predicted roles such as electron transport, membrane trafficking etc. | NDUFAF2, PTGES2, ACE, and COMT | SARS-CoV-2 | (Gordon et al., 2020) |
| Nsp8 | Predicted role in regulating mitochondrial ribosomal pathway. | MRPS proteins | SARS-CoV-2 | (Gordon et al., 2020) |
| Nsp9 | Predicted role in regulating nuclear pore transport and translation pathway. | NUP proteins, EIF4H, and NEK9 | SARS-CoV-2 | (Gordon et al., 2020) |
| Nsp12 | Probable role in apoptotic and necroptotic cell death pathway. | RIPK1, etc. | SARS-CoV-2 | (Gordon et al., 2020) |
| Nsp13 | Predicted role in transcriptional regulation, protein kinase A signaling, golgi organisation, centrosome organisation, and host cell translation. | PRKACA, TBK1, etc. | SARS-CoV-2 | (Gordon et al., 2020) |
| Nsp14 | The N7-methylguanosine (m7G) cap | Viral RNA | SARS CoV | (Chen et al., 2009) |
|  | Probable role in nucleic acid synthesis pathway. | GLA, IMPDH2, etc. | SARS-CoV-2 | (Gordon et al., 2020) |
| Nsp15 | Inhibits IFN-β signaling | Unexplored | SARS CoV | (Frieman et al., 2009) |
| Nsp16 | Involved in Ribose 2′-O-methylation at the 5’ end of the RNA to prevent sensing by MDA5 | MDA5 | SARS CoV | (Züst et al., 2011) |
| S protein | Inhibits host cell translation by interacting and inhibiting translation initiation factor eIF3f | eIF3f | SARS-CoV | (Xiao et al., 2008) |
|  | Induces apoptosis in Vero E6 cells | Not known |  | (Chow et al., 2008) |
| ORF3a | Induces apoptosis of cells by causing fragmentation of golgi apparatus and vesicle formation. | Multiple  Golgi apparatus  vesicles | SARS CoV | (Waye et al., 2005; Freundt et al., 2010) |
|  | Overexpression of ORF3a induced apoptosis in lung epithelial cells. | Unexplored |  | (Law et al., 2005) |
|  | Leads to cell death by necroptosis by interacting with and activating RIPK3. | RIPK3 |  | (Yue et al., 2018) |
|  | Plasmid harboring ORF3a induces apoptosis in HEKT93 cells when overexpressed. | Caspase 8 | SARS-CoV-2 | (Ren et al., 2020) |
| ORF3b | Inhibits phosphorylation of IRF3 | IRF3 | SARS CoV | (Freundt et al., 2009) |
|  | Not explored, but SARS-CoV-2 has comparably short ORF3b with no homology to SARS CoV. | Not known | SARS-CoV-2 | (Mantlo et al., 2020) |
|  | Induces apoptosis and necrosis in cells overexpressing this protein. | Unexplored | SARS-CoV | (Khan et al., 2006) |
|  | Induces apoptosis by causing G0/G1 cell cycle arrest. | Possibly interfering with cell cycle | SARS-CoV | (Yuan et al., 2005) |
|  | The anti-IFN response of ORF3b was shown to be higher in SARS-CoV-2 than SARS-CoV. | Unexplored | SARS-CoV-2 | (Gordon et al., 2020) |
| E protein | Predicted role in regulating Bromodomain-containing proteins which are implicated in regulating antiviral response by regulating gene transcription. | BRD2, BRD4 | SARS-CoV-2 | (Gordon et al., 2020) |
| M protein | Inhibits NAS signaling by directly interacting with RIG-I, TBK1, IKKϵ, and TRAF3 complex. | RIG-I, TBK1, IKKϵ, and TRAF3 | SARS-CoV | (Siu et al., 2009) |
|  | Modulates AKT pathway and causes the release of cytochrome c from mitochondria to induce apoptosis | AKT | SARS-CoV | (Chan et al., 2007) |
|  | Multiple predicted roles like on mitochondrial metabolism, RNA processing, nuclear pore, electron transport chain, etc. | ABCC1, etc. | SARS-CoV-2 | (Gordon et al., 2020) |
| ORF6 | Inhibits phosphorylation of IRF3. | IRF3 | SARS-CoV | (Kopecky-Bromberg et al., 2007) |
|  | Interacts with karyopherin α2 and karyopherin β1 and disrupts nuclear import which then prevents translocation of STAT1 to the nucleus. | karyopherin α2 karyopherin β1 |  | (Frieman et al., 2007) |
|  | Inhibits IFN-β, NF-κB and ISRE response in HEK-293 T. | Not known | SARS-CoV-2 | (Li et al., 2020b) |
|  | Predicted role in regulating nuclear pore. | NUP98, RAE1, DCTPP1, | SARS-CoV-2 |  |
| ORF7a | Inhibits host cell protein synthesis by activating MAPK signaling. | MAPK | SARS CoV | (Kopecky-Bromberg et al., 2006) |
|  | Interacts with Bcl-X_L_ and induces apoptosis. | Bcl-X_L_ |  | (Tan et al., 2007) |
| ORF8a | Induces mitochondrial membrane depolarization and ROS mediated cellular apoptosis. | Mitochondria |  | (Chen et al., 2007) |
| ORF8b  ORF8ab | Directly interacts with IRF3 and induces its degradation in a ubiquitin-dependent manner. | IRF3 | SARS CoV | (Wong et al., 2018) |
| ORF8b | ORF8b forms aggregates which induce mitochondrial, ER, and lysosomal dysfunction followed by cell death. | NLRP3 | SARS CoV | (Shi et al., 2019) |
|  | SARS-CoV-2 lack a functional motif which was previously shown to activate NLRP3 pathway and cell death. Further, ORF8b of CoV-2 is a secretory protein unlike CoV-1. | Not known | SARS-CoV-2 | (Chan et al., 2020) |
| ORF8 | Inhibits IFN-β, NF-κB and ISRE response in HEK-293 T. | Not known | SARS-CoV-2 | (Li et al., 2020b) |
|  | Predicted role in regulating glycosylation, ER protein quality control, and glycosaminoglycan synthesis. | DNMT1, LOX, FKBP7/10, etc. | SARS-CoV-2 | (Gordon et al., 2020) |
| N protein | Blocks association of TRIM-25 with RIG-1 | TRIM-25 | SARS-CoV | (Hu et al., 2017) |
|  | Inhibits phosphorylation of IRF3 and thus antagonizes IFN signaling. | IRF3 | SARS-CoV | (Kopecky-Bromberg et al., 2007) |
|  | Inhibits IFN-β, NF-κB response in HEK-293 T. | Not known | SARS-CoV-2 | (Li et al., 2020b) |
|  | Predicted role in preventing stress granule formation and thereby interfering with antiviral response. Further, role in host cell RNA processing is also predicted. | CSNK2B, CSNK2A2, and LARP1. | SARS-CoV-2 | (Gordon et al., 2020) |
| ORF9b | Induces proteasomal degradation of mitochondrial DRP1, MAVS, and TRAF3/6 by associating these proteins with PCBP2 and the HECT domain E3 ligase AIP4 to induce their ubiquitin mediated degradation. | DRP1, MAVS, PCBP2 and AIP4 ligase | SARS CoV | (Shi et al., 2014) |
|  | Interacts with the cellular protein Crm1 and induces host cell apoptosis. | Crm1 |  | (Sharma et al., 2011) |
|  | Predicted role in regulating MARK kinase signaling. | MARK proteins and DCTPP1. | SARS-CoV-2 | (Gordon et al., 2020) |
| ORF9c | Predicted role in mitochondrial electron transport chain. | NDUFAF1, NDUFB9, ABCC1, F2RL1, Sigma-2 receptor. | SARS-CoV-2 | (Gordon et al., 2020) |
| ORF10 | Predicted role in regulating ubiquitination-dependent protein degradation of the host cell proteins | CUL2, ELOB, ELOC, RBX1, ZYG11B, |  | (Gordon et al., 2020) |

**Abbreviations:**

*3CL^pro^: 3C-like protease; ABCC1: ATP Binding Cassette Subfamily C Member 1; ACE: Angiotensin-converting enzyme; ATP6AP1: ATPase H+ Transporting Accessory Protein 1; BRD: Bromodomain-containing protein; Crm1: Chromosomal Maintenance 1, also known as Exportin 1; COMT: Catechol-O-methyltransferase; CSNK: Casein kinase I gamma; CUL2: Cullin-2; DCTPP1: dCTP pyrophosphatase 1; DNMT1: DNA (cytosine-5)-methyltransferase 1; Dynamin related protein 1; ELF4E: Eukaryotic translation initiation factor 4E; DRP1; PLpro: Papain-like protease; EIF4H: Eukaryotic translation initiation factor 4H; ELOB: Transcription elongation factor B polypeptide 2; ELOC: Transcription elongation factor B polypeptide 1FKBP: FK506-binding proteins; F2RL1: F2R Like Trypsin Receptor 1; HDAC2: Histone deacetylase 2; ISRE; Interferon-stimulated response element; LARP1: La Ribonucleoprotein 1, Translational Regulator; LOX: Lysyl oxidase; MAPK: activated p38 mitogen-activated protein kinase; MARK: MAP/microtubule affinity-regulating kinase 4; MRPS: multidrug resistance proteins; NDUFAF: NADH Ubiquinone; Oxidoreductase Complex Assembly Factor; Nsp: Non-structural protein; NUP: Nucleoporins proteins; NUP210: Nuclear pore membrane glycoprotein 210; NEK9: NimA (never in mitosis A) family of serine/ threonine protein kinases; ORF: Open reading frame; PCB2: Poly (rC)-binding protein 2; PRKACA: Protein kinase A catalytic subunit; PTGES2: Prostaglandin E Synthase 2 RBX1: Ring-Box 1; RCHY1: RING finger and CHY zinc finger domain-containing protein; RIPK1: Receptor-interacting serine/threonine-protein kinase 1; ZYG11B: zyg-11 homolog B.*

Boxes with light green color indicate host cell target of SARS-CoV-2.

**REFERENCES**

Chan, C. M., Ma, C. W., Chan, W. Y., and Chan, H. Y. E. (2007). The SARS-Coronavirus Membrane protein induces apoptosis through modulating the Akt survival pathway. *Arch. Biochem. Biophys.* 459, 197–207. doi:10.1016/j.abb.2007.01.012.

Chan, J. F. W., Yuan, S., Kok, K. H., To, K. K. W., Chu, H., Yang, J., et al. (2020). A familial cluster of pneumonia associated with the 2019 novel coronavirus indicating person-to-person transmission: a study of a family cluster. *Lancet.* 395, 514–523. doi:10.1016/S0140-6736(20)30154-9.

Chen, C. Y., Ping, Y. H., Lee, H. C., Chen, K. H., Lee, Y. M., Chan, Y. J., et al. (2007). Open reading frame 8a of the human severe acute respiratory syndrome coronavirus not only promotes viral replication but also induces apoptosis. *J. Infect. Dis.* 196, 405–415. doi:10.1086/519166.

Chen, X., Yang, X., Zheng, Y., Yang, Y., Xing, Y., and Chen, Z. (2014). SARS coronavirus papain-like protease inhibits the type I interferon signaling pathway through interaction with the STING-TRAF3-TBK1 complex. *Protein Cell.* 5, 369–381. doi:10.1007/s13238-014-0026-3.

Chen, Y., Cai, H., Pan, J., Xiang, N., Tien, P., Ahola, T., et al. (2009). Functional screen reveals SARS coronavirus nonstructural protein nsp14 as a novel cap N7 methyltransferase. *Proc. Natl. Acad. Sci. U. S. A.* 106, 3484–3489. doi:10.1073/pnas.0808790106.

Chow, K. Y., Yeung, Y. S., Hon, C. C., Zeng, F., Law, K. M., and Leung, F. C. C. (2008). SARS coronavirus and apoptosis. *Hong Kong Med. J.* 14, 8–13.

Devaraj, S. G., Wang, N., Chen, Z., Chen, Z., Tseng, M., Barretto, N., et al. (2007). Regulation of IRF-3-dependent innate immunity by the papain-like protease domain of the severe acute respiratory syndrome coronavirus. *J. Biol. Chem.* 282, 32208–32221. doi:10.1074/jbc.M704870200.

Freundt, E. C., Yu, L., Goldsmith, C. S., Welsh, S., Cheng, A., Yount, B., et al. (2010). The Open Reading Frame 3a Protein of Severe Acute Respiratory Syndrome-Associated Coronavirus Promotes Membrane Rearrangement and Cell Death. *J. Virol.* 84, 1097–1109. doi:10.1128/jvi.01662-09.

Frieman, M., Ratia, K., Johnston, R. E., Mesecar, A. D., and Baric, R. S. (2009). Severe Acute Respiratory Syndrome Coronavirus Papain-Like Protease Ubiquitin-Like Domain and Catalytic Domain Regulate Antagonism of IRF3 and NF-κB Signaling. *J. Virol.* 83, 6689–6705. doi:10.1128/jvi.02220-08.

Frieman, M., Yount, B., Heise, M., Kopecky-Bromberg, S. A., Palese, P., and Baric, R. S. (2007). Severe Acute Respiratory Syndrome Coronavirus ORF6 Antagonizes STAT1 Function by Sequestering Nuclear Import Factors on the Rough Endoplasmic Reticulum/Golgi Membrane. *J. Virol.* 81, 9812–9824. doi:10.1128/jvi.01012-07.

Gordon, D. E., Jang, G. M., Bouhaddou, M., Xu, J., Obernier, K., White, K. M., et al. (2020). A SARS-CoV-2 protein interaction map reveals targets for drug repurposing. *Nature.* 583, 459–468. doi:10.1038/s41586-020-2286-9.

Hu, Y., Li, W., Gao, T., Cui, Y., Jin, Y., Li, P., et al. (2017). The Severe Acute Respiratory Syndrome Coronavirus Nucleocapsid Inhibits Type I Interferon Production by Interfering with TRIM25-Mediated RIG-I Ubiquitination. *J. Virol.* 91. doi:10.1128/jvi.02143-16.

Huang, C., Lokugamage, K. G., Rozovics, J. M., Narayanan, K., Semler, B. L., and Makino, S. (2011). SARS coronavirus nsp1 protein induces template-dependent endonucleolytic cleavage of mRNAs: Viral mRNAs are resistant to nsp1-induced RNA cleavage. *PLoS Pathog.* 7. doi:10.1371/journal.ppat.1002433.

Khan, S., Fielding, B. C., Tan, T. H. P., Chou, C. F., Shen, S., Lim, S. G., et al. (2006). Over-expression of severe acute respiratory syndrome coronavirus 3b protein induces both apoptosis and necrosis in Vero E6 cells. *Virus Res.* 122, 20–27. doi:10.1016/j.virusres.2006.06.005.

Kopecky-Bromberg, S. A., Martinez-Sobrido, L., and Palese, P. (2006). 7a Protein of Severe Acute Respiratory Syndrome Coronavirus Inhibits Cellular Protein Synthesis and Activates p38 Mitogen-Activated Protein Kinase. *J. Virol.* 80, 785–793. doi:10.1128/jvi.80.2.785-793.2006.

Law, H. K. W., Chung, Y. C., Hoi, Y. N., Sin, F. S., Yuk, O. C., Luk, W., et al. (2005). Chemokine up-regulation in SARS-coronavirus-infected, monocyte-derived human dendritic cells. *Blood.* 106, 2366–2374. doi:10.1182/blood-2004-10-4166.

Li, J. Y., Liao, C. H., Wang, Q., Tan, Y. J., Luo, R., Qiu, Y., et al. (2020). The ORF6, ORF8 and nucleocapsid proteins of SARS-CoV-2 inhibit type I interferon signaling pathway. *Virus Res.* 286. doi:10.1016/j.virusres.2020.198074.

Ma-Lauer, Y., Carbajo-Lozoya, J., Hein, M. Y., Müller, M. A., Deng, W., Lei, J., et al. (2016). P53 down-regulates SARS coronavirus replication and is targeted by the SARS-unique domain and PLpro via E3 ubiquitin ligase RCHY1. *Proc. Natl. Acad. Sci. U. S. A.* 113, E5192–E5201. doi:10.1073/pnas.1603435113.

Mantlo, E., Bukreyeva, N., Maruyama, J., Paessler, S., and Huang, C. (2020). Antiviral activities of type I interferons to SARS-CoV-2 infection. *Antiviral Res.* 179. doi:10.1016/j.antiviral.2020.104811.

Narayanan, K., Huang, C., Lokugamage, K., Kamitani, W., Ikegami, T., Tseng, C.-T. K., et al. (2008). Severe Acute Respiratory Syndrome Coronavirus nsp1 Suppresses Host Gene Expression, Including That of Type I Interferon, in Infected Cells. *J. Virol.* 82, 4471–4479. doi:10.1128/jvi.02472-07.

Ren, Y., Shu, T., Wu, D., Mu, J., Wang, C., Huang, M., et al. (2020). The ORF3a protein of SARS-CoV-2 induces apoptosis in cells. *Cell. Mol. Immunol.* 17, 881–883. doi:10.1038/s41423-020-0485-9.

Sharma, K., Åkerström, S., Sharma, A. K., Chow, V. T. K., Teow, S., Abrenica, B., et al. (2011). SARS-CoV 9b protein diffuses into nucleus, undergoes active Crm1 mediated nucleocytoplasmic export and triggers apoptosis when retained in the nucleus. *PLoS One.* 6. doi:10.1371/journal.pone.0019436.

Siu, K. L., Kok, K. H., Ng, M. H. J., Poon, V. K. M., Yuen, K. Y., Zheng, B. J., et al. (2009). Severe acute respiratory syndrome coronavirus M protein inhibits type I interferon production by impeding theformation of TRAF3·TANK·TBK1/IKKε complex. *J. Biol. Chem.* 284, 16202–16209. doi:10.1074/jbc.M109.008227.

Tanaka, T., Kamitani, W., DeDiego, M. L., Enjuanes, L., and Matsuura, Y. (2012). Severe Acute Respiratory Syndrome Coronavirus nsp1 Facilitates Efficient Propagation in Cells through a Specific Translational Shutoff of Host mRNA. *J. Virol.* 86, 11128–11137. doi:10.1128/jvi.01700-12.

Wathelet, M. G., Orr, M., Frieman, M. B., and Baric, R. S. (2007). Severe Acute Respiratory Syndrome Coronavirus Evades Antiviral Signaling: Role of nsp1 and Rational Design of an Attenuated Strain. *J. Virol.* 81, 11620–11633. doi:10.1128/jvi.00702-07.

Waye, M. M. Y., Law, P. T. W., Wong, C. H., Au, T. C. C., Chuck, C. P., Kong, S. K., et al. (2005). The 3a protein of SARS-coronavirus induces apoptosis in vero E6 cells. in *Annual International Conference of the IEEE Engineering in Medicine and Biology - Proceedings*, 7482–7485. doi:10.1109/iembs.2005.1616242.

Wong, H. H., Fung, T. S., Fang, S., Huang, M., Le, M. T., and Liu, D. X. (2018). Accessory proteins 8b and 8ab of severe acute respiratory syndrome coronavirus suppress the interferon signaling pathway by mediating ubiquitin-dependent rapid degradation of interferon regulatory factor 3. *Virology.* 515, 165–175. doi:10.1016/j.virol.2017.12.028.

Xiao, H., Xu, L. H., Yamada, Y., and Liu, D. X. (2008). Coronavirus spike protein inhibits host cell translation by interaction with eIF3f. *PLoS One* 3. doi:10.1371/journal.pone.0001494.

Yuan, L., Chen, Z., Song, S., Wang, S., Tian, C., Xing, G., et al. (2015). P53 degradation by a coronavirus papain-like protease suppresses type I interferon signaling. *J. Biol. Chem.* 290, 3172–3182. doi:10.1074/jbc.M114.619890.

Yuan, X., Shan, Y., Zhao, Z., Chen, J., and Cong, Y. (2005). G0/G1 arrest and apoptosis induced by SARS-CoV 3b protein in transfected cells. *Virol. J.* 2. doi:10.1186/1743-422X-2-66.

Yue, Y., Nabar, N. R., Shi, C. S., Kamenyeva, O., Xiao, X., Hwang, I. Y., et al. (2018). SARS-Coronavirus Open Reading Frame-3a drives multimodal necrotic cell death. *Cell Death Dis.* 9. doi:10.1038/s41419-018-0917-y.

Züst, R., Cervantes-Barragan, L., Habjan, M., Maier, R., Neuman, B. W., Ziebuhr, J., et al. (2011). Ribose 2’-O-methylation provides a molecular signature for the distinction of self and non-self mRNA dependent on the RNA sensor Mda5. *Nat. Immunol.* 12, 137–143. doi:10.1038/ni.1979.
